# Prion Protein Octarepeat Domain Forms Transient β-Sheet Structures upon Residue-Specific Binding to Cu(II) and Zn(II) Ions

**DOI:** 10.1021/acs.biochem.3c00129

**Published:** 2023-05-10

**Authors:** Maciej Gielnik, Aneta Szymańska, Xiaolin Dong, Jüri Jarvet, Željko M. Svedružić, Astrid Gräslund, Maciej Kozak, Sebastian K. T. S. Wärmländer

**Affiliations:** † Department of Macromolecular Physics, Faculty of Physics, Adam Mickiewicz University, PL 61-614 Poznań, Poland; ‡ Department of Biomedical Chemistry, Faculty of Chemistry, Gdańsk University, PL 80-308 Gdańsk, Poland; § Chemistry Section, Stockholm University, 10691 Stockholm, Sweden; ∥ The National Institute of Chemical Physics and Biophysics, 12618 Tallinn, Estonia; ⊥ Department of Biotechnology, University of Rijeka, HR 51000 Rijeka, Croatia; # National Synchrotron Radiation Centre SOLARIS, Jagiellonian University, PL 30-392 Kraków, Poland

## Abstract

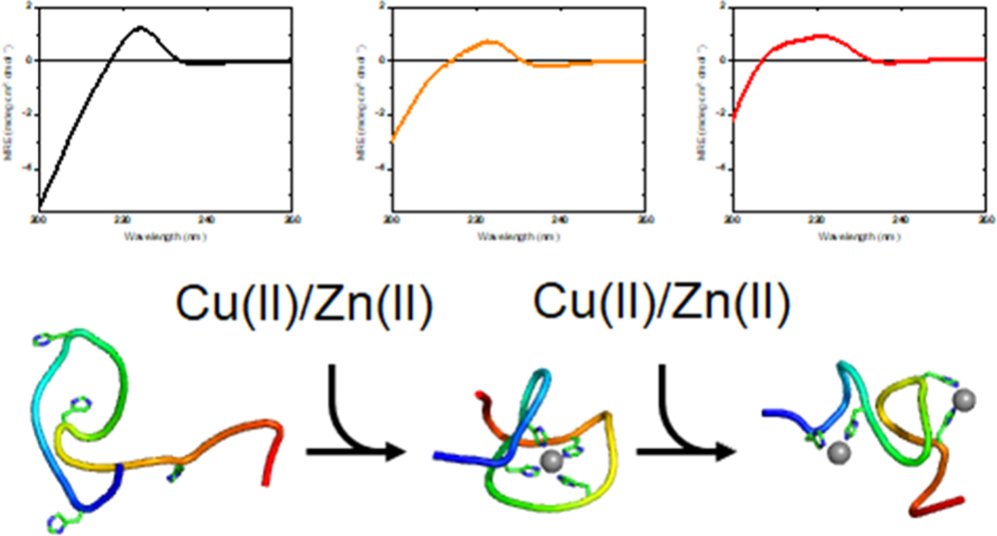

Misfolding of the cellular prion protein (PrP^C^) is associated with the development of fatal neurodegenerative diseases called transmissible spongiform encephalopathies (TSEs). Metal ions appear to play a crucial role in PrP^C^ misfolding. PrP^C^ is a combined Cu(II) and Zn(II) metal-binding protein, where the main metal-binding site is located in the octarepeat (OR) region. Thus, the biological function of PrP^C^ may involve the transport of divalent metal ions across membranes or buffering concentrations of divalent metal ions in the synaptic cleft. Recent studies have shown that an excess of Cu(II) ions can result in PrP^C^ instability, oligomerization, and/or neuroinflammation. Here, we have used biophysical methods to characterize Cu(II) and Zn(II) binding to the isolated OR region of PrP^C^. Circular dichroism (CD) spectroscopy data suggest that the OR domain binds up to four Cu(II) ions or two Zn(II) ions. Binding of the first metal ion results in a structural transition from the polyproline II helix to the β-turn structure, while the binding of additional metal ions induces the formation of β-sheet structures. Fluorescence spectroscopy data indicate that the OR region can bind both Cu(II) and Zn(II) ions at neutral pH, but under acidic conditions, it binds only Cu(II) ions. Molecular dynamics simulations suggest that binding of either metal ion to the OR region results in the formation of β-hairpin structures. As the formation of β-sheet structures can be a first step toward amyloid formation, we propose that high concentrations of either Cu(II) or Zn(II) ions may have a pro-amyloid effect in TSE diseases.

## 1 Introduction

Transmissible spongiform encephalopathies (TSEs) are a group of neurodegenerative disorders initiated by misfolding of the cellular prion protein (PrP^C^).^1,2^ The human PrP^C^ is a 208-residue-long protein expressed at a high level in the central nervous system. It is composed of two structurally different regions: an unstructured N-terminal domain and a globular and mostly α-helical C-terminal domain^3^ that attaches to the pre- and postsynaptic membranes via a GPI anchor.^4,5^ For unknown reasons, PrP^C^ can undergo a structural transition into PrP^Sc^, an insoluble, aggregated form with high amounts of β-sheet secondary structure.^1,2^ Even though human TSEs are very rare and only affect one person per million,^6^ they share many similarities with the pathologies characterized by protein aggregating into amyloid states. In fact, multiple pieces of evidence indicate that all of the prion and amyloid diseases belong to a large family of protein aggregation diseases.^7−10^ Examples include tauopathies (tau protein),^11^ Alzheimer’s disease (amyloid-β peptide),^12^ Parkinson’s disease (α-synuclein protein),^13^ and amyotrophic lateral sclerosis/ALS (TDP-43 protein).^14,15^ Thus, it has recently been proposed that besides TSEs, PrP can also be involved in the development of other neurodegenerative diseases, such as Alzheimer’s disease.^16^ However, unlike most amyloid diseases, TSEs can sometimes be transmitted between species.^2^ It has been suggested that PrP is most toxic when forming soluble oligomers, which can accumulate in brain tissue and cause neurodegeneration.^17^ Similar to oligomers of tau, amyloid-β, and α-synuclein proteins, such PrP oligomers often display higher β-sheet content than the corresponding monomers.^18−22^

The native function of PrP^C^ is still elusive. The PrP^C^ protein is encoded by the *PRNP* gene, and most *PRNP* knockout animals (i.e., without the *PRNP* gene) show normal development and behavior, although some individuals show deviation in neuronal signal transduction and locomotion.^23^ Interestingly, all *PRNP* knockout mice are immune to PrP^Sc^ inoculation, which supports the theory of template-driven autocatalytic conversion of PrP^C^ to PrP^Sc 2,8^. Among the many functions attributed to PrP^C^, i.e., cell signaling, antioxidation, and myelination,^17^ phylogenetic analysis indicates that PrP^C^ is evolutionarily linked to the Zrt- and Irt-like protein (ZIP) family of divalent metal ion transporters.^24^ This suggests that one biological role for PrP^C^ might be to regulate metal ion homeostasis—metal imbalance has been suggested to be part of the pathology of prion diseases.^25−27^

**Figure 1:**
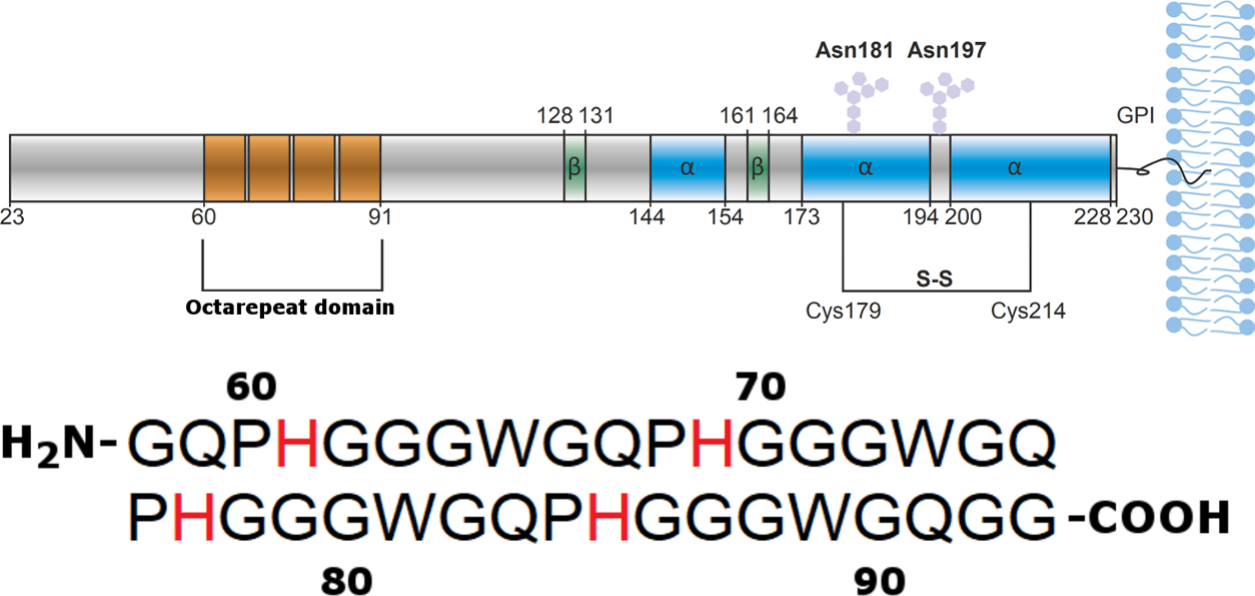
Top: sequence of the human prion protein, with the octarepeat region marked in orange, β-sheets marked in green, and α-helices marked in blue. The image is from Gielnik et al.^28^ under a CC BY 4.0 license. Bottom: the octarepeat (OR) region studied in this paper comprises residues 58–93 of the prion protein, i.e., PrP^C^(58–93). At neutral pH, it has no charged residues. Possible metal-binding aromatic histidine residues are shown in red.

The PrP^C^ protein is known to bind up to six different types of divalent metal ions, including Cu(II), Zn(II), Ni(II), and Mn(II), by two distinct domains with different metal ion affinities.^29−31^ The octarepeat (OR) region is located in the N-terminal domain, where it spans residues 60–91 (Figure 1). It contains four tandem PHGGGWGQ repeats and binds Cu(II), Zn(II), and Ni(II) ions with strong affinity (around 0.1 nM for Cu(II),^32^ 10 nM for Ni(II), and 400 nM for Zn(II)^33^). The so-called “non-octarepeat region” spans residues 92–111 and binds Cu(II) ions with weaker affinity, where His96 and His111 are likely binding ligands.^34,35^ The capacity of the OR region to bind Cu(II) ions has been intensively studied during the last twenty years. Cu(II) is an important neurotransmitter and the third most common transition metal in the brain.^36^ The reported Cu(II) concentration in the synaptic cleft during neuron depolarization ranges from 3^37^ to 250 μM,^38^ which suggests that the dissociation constant (*K*_d_) for the OR·Cu(II) complex is at least of this order of magnitude. The OR region has been reported to bind up to four Cu(II) ions,^39−41^ where the first ion binds with the highest affinity (around 0.1 nM),^32^ the second with moderate affinity (around 200 nM),^42^ and the third and the fourth Cu(II) ions with weaker affinities (around 10 μM).^32^ Many possible biological functions have been attributed to the Cu(II)-binding capacity of PrP^C^, including superoxide dismutase activity, transmembrane copper transport, copper buffering, and neuronal protection.^43−46^

Another important metal ion for PrP^C^ neurobiology is Zn(II). Zinc is the second-most abundant (after iron) transition metal ion in the human body.^47^ Upon neuronal stimulation, the transient concentration of Zn(II) ions in the synaptic vesicle can reach values around 300 μM.^48^ Such Zn(II) concentrations stimulate PrP^C^ endocytosis into human neuroblastoma cells,^49^ and PrP^C^ has been shown to enhance Zn(II) transport.^50^ An early study reported a *K*_d_ of 200 μM for the PrP^C^·Zn(II) complex,^51^ but more recent isothermal titration calorimetry (ITC) studies by the same researchers suggest a *K*_d_ of 17 μM, together with NMR experiments implying 1:1 stoichiometry.^52^ The proposed PrP^C^ functions related to Zn(II) binding are similar to those proposed for Cu(II) binding, e.g., metal ion buffering and transport.^27,53^

We have recently shown that the isolated OR region (i.e., an OR peptide), upon interaction with Zn(II) ions, forms fibrillar cross-β structures that bind thioflavin T and Congo red and which possess all of the characteristic features of the amyloid material.^54^ In vivo, PrP^C^ undergoes α-cleavage at residues Lys110–His111 or His111–Met112, and β-cleavage at residues Gln91–Glu92, yielding N-terminal fragments that include the OR sequence.^55−57^ For such fragments, bound metal ions may induce aggregation into amyloid states. Some studies have shown that the N-terminal domain, including the OR region, is essential for PrP dimerization, where Cu(II) ions promote the formation of dimers as well as further PrP aggregation.^58^

Here, we use circular dichroism (CD) and fluorescence spectroscopy, combined with molecular dynamics simulations, to estimate apparent *K*_d_ values for the OR·Cu(II) and OR·Zn(II) complexes and to characterize the initial structural changes of the isolated OR region, i.e., PrP^C^(58–93) (Figure 1), after exposure to Cu(II) and Zn(II) ions.

## 2 Materials and Methods

### 2.1 Peptide Synthesis and Purification

The OR peptide, i.e., fragment 58–93 of the human prion protein (UniProt ID P04156), was produced via solid peptide synthesis. TentaGel R RAM resin (loading capacity of 0.18 mmol/g; Rapp Polymere, Germany) was used as a matrix. The synthesis was performed using a standard Fmoc/tBu amino acid chemistry on a Liberty Blue (CEM Corp.) microwave peptide synthesizer. The crude peptide was cleaved from the solid support using a cleavage cocktail consisting of 88% trifluoroacetic acid (TFA), 5% water, 5% phenol, and 2% triisopropylsilane (v/v/m/v) under a neutral (argon) atmosphere, and protected from direct exposure to light. After precipitation with diethyl ether, the crude product was dissolved in water and lyophilized.

Peptide purification was carried out by reversed-phase high-performance liquid chromatography (RP-HPLC) using a Luna C8(2) AXIA Pack column (250 × 21.2 mm^3^, 5 μm, 100 Å; Phenomenex Inc.). A linear gradient of acetonitrile in 0.1% aqueous TFA was applied as a mobile phase. The purity of the obtained fractions was evaluated by analytical UHPLC, using a Kinetex C8 column (100 × 2.1 mm^2^, 2.6 μm, 100 Å; Phenomenex Inc.) operated by the NEXERA-i chromatography system (Shimadzu, Japan) in a 15 min linear gradient of 5–100% B (where B is 80% acetonitrile in 0.1% aqueous TFA). UV absorption was monitored at λ = 223 nm. Fractions with a purity higher than 99% were pooled together for further analysis. The molecular weight of the final peptide sample was confirmed by mass spectrometry using an ESI-IT-TOF-LC-MS system (Shimadzu, Japan) with a C12 Jupiter Proteo column (150 × 2 mm^2^, 4 μm, 90 Å; Phenomenex Inc.).

### 2.2 Circular Dichroism Spectroscopy

Circular dichroism (CD) spectra of the OR peptide were recorded on a Chirascan CD (Applied Photophysics, U.K.) spectropolarimeter. Thermal unfolding experiments were performed for 20 μM OR peptide in 10 mM sodium phosphate buffer, pH 7.0, in a cuvette with a 4 mm path length, in the range from 5 to 65 °C with 5 °C intervals. These spectra were recorded from 190 to 250 nm, with 0.5 nm steps and a time-per-point of 4 s. The content of the polyproline II helix (PPII) helix was estimated from the CD intensity expressed in mean residue ellipticity (a concentration-independent unit) at the local maximum at 225 nm of the CD spectra, i.e., θ_max_, using the equation published by Kelly et al.,^59^ i.e., eq 1
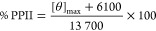
1Titrations with metal ions were conducted for 5 μM OR peptide dissolved either in pure Milli-Q water or in 10 mM sodium phosphate buffer, pH 7.5. Using 1 cm path-length quartz cuvettes with gentle magnetic stirring at 25 °C, the OR peptide (volume 2.5 mL) was titrated with small amounts of stock solutions of CuCl_2_ or ZnCl_2_ (100 μM, 500 μM, 1.25 mM, or 5 mM stock concentrations) directly in the cuvette and the samples were equilibrated for 10 min. All spectra were collected from 200 to 260 nm with sampling points every 0.5 nm, a time-per-point of 4 s, and 2 nm bandwidth. This resulted in a scan time of over 8 min for each spectrum. The total time difference between measurements at the same wavelength was therefore around 18 min, which should be sufficient for the sample to reach equilibrium. The final spectra were baseline-corrected and smoothed with a Savitzky–Golay filter. Data with single visible transitions were fitted to the transformed Hill equation, i.e., eq 2
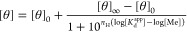
2Here, [θ]_0_ is the signal intensity before the transition, [θ]_∞_ is the signal intensity at the end of the transition, *n*_H_ is the Hill coefficient, [*K*_d_^app^] is the apparent dissociation constant, and [Me] is the metal ion concentration. When two transitions were observed, and the signal was monotonically increasing or decreasing, the data was fitted as a sum of two transformed Hill equations, i.e., eq 3
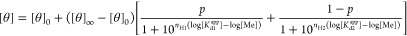
3Here, [θ]_0_ is the signal intensity before the transition, [θ]_∞_ is the signal intensity at saturation, [*K*_d1_^app^] and [*K*_d2_^app^] are the apparent dissociation constants for the first and second binding sites, respectively, *n*_H1_ and *n*_H2_ are the Hill coefficients for the first and second binding sites, respectively, [Me] is the metal ion concentration, and *p* and 1 – *p* are the relative signal intensities for the first and second binding sites, respectively, obtained from the fit, but with initial values extracted from the data. When the two transitions were observed with a local maximum or minimum in the signal, data was fitted as the sum of one transformed and one reverse-transformed Hill equation, i.e., eq 4
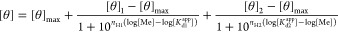
4Here, [θ]_max_ is the signal intensity at the extreme point, [θ]_1_ is the signal intensity before the transition, [θ]_2_ is the signal intensity after the transition, and *n*_H1_, *n*_H2_, [*K*_d1_^app^], [*K*_d2_^app^], and [Me] have the same meaning as in eq 3. The data were fitted as a sum of Hill equations because 5 μM OR peptide with *K*_d1_^real^ ∼ 0.1 nM is fully saturated at 5 μM of metal ion and 5 μM OR peptide with *K*_d2_^real^ ∼ 200 nM is fully saturated at 10 μM of metal ion.^42^

### 2.3 Fluorescence Spectroscopy

Five micromolar OR peptide was titrated with stock solutions of CuCl_2_ and ZnCl_2_, similar to the CD titrations (above), and using the following different buffers: (i) 10 mM sodium phosphate buffer, pH 7.5, (ii) 10 mM 2-morpholinoethanesulfonic acid (MES) buffer, pH 5.5, and (iii) 10 mM MES buffer, pH 7.5. In order to investigate the effect of added Cu(I) ions, some titrations were performed in the presence of 1 mM tris(2-carboxyethyl)phosphine (TCEP), a well-known reducing agent. To remove molecular oxygen from the samples with TCEP, these samples were bubbled for 10 min with gaseous nitrogen. Fluorescence spectra of the OR peptide were recorded with a Cary Eclipse (Varian) fluorometer equipped with a Peltier multicell holder, using quartz cuvettes with 1 cm path length. The samples were excited at 285 nm, and emission spectra were recorded in the 300–500 nm range, with a 1 nm data interval and a scan speed of 600 nm/min. The excitation and emission bandwidths were 10 and 5 nm, respectively, and the experiments were performed at 25 °C under quiescent conditions (i.e., no stirring). The fluorescence intensity at 355 nm was plotted versus metal ion concentration, and the resulting data curves were fitted with the same equations as those used for CD data (i.e., eq 2).

### 2.4 Molecular Dynamics Simulations

Molecular dynamics simulations were performed in GROMACS 2019.2^60^ using the OPLS-AA^61^ force field. The systems were solvated with the TIP4P^62^ water model and restrained using van der Waals radii.^63^ The LINCS algorithm^64^ was used to restrain all covalent bonds in the peptide, and the SETTLE algorithm^65^ was used to restrain all water molecules. In the initial mode, one or two nonbonded dummy Cu(II) or Zn(II) ion models^66^ were placed in close proximity to N^ε2^ atoms of histidine side chains. The N^ε2^ atoms of the four histidine residues were either protonated (positively charged) or deprotonated (neutral) to compare the interactions under different pH conditions (for short peptides, histidine protonation/deprotonation corresponds to pH < 6.8 or >6.8, respectively).^67^ The systems were neutralized with Cl^–^ ions, and the energy was minimized using steepest-descent energy minimization over 5000 steps. The temperature was equilibrated at 300 K in a canonical (NVT) ensemble over 0.5 ns with 1 fs time steps using the modified Berendsen^68^ thermostat. The pressure was equilibrated at 1 bar in an isothermal-isobaric (NpT) ensemble over 0.5 ns with 1 fs time steps using the Parrinello–Rahman^69^ barostat. For long-range electrostatic interactions, we applied PME^70^ with 1.2 nm cutoff, and the same cutoff was used for van der Waal forces. The molecular dynamics (MD) production runs were performed in an NVT ensemble with 2 fs time steps. The trajectories for the peptide with neutral histidine residues were produced over 100 ns, while trajectories for the peptide with positively charged histidine residues were produced over 10 ns. The results were analyzed in VMD^71^ and visualized in the PyMOL Molecular Graphics System, version 2.3.4 (Schrödinger, LLC). The secondary structure was assigned using the PROSS software,^72^ which estimates secondary structures as α-helix, β-strand, β-turn, and polyproline II helix (PPII), and classifies all other structures as the “coil”. Principal component analysis (PCA) was performed using the Bio3D R package.^73^

### 2.5 Calculation of p*K*_a_ Values

Final peptide conformations were extracted as frames from MD simulations by storing the structures as PDB files, which were then used as input for the two best-known protocols for p*K*_a_ calculations for protein structures. Thus, p*K*_a_ values for the OR peptide were calculated using both PropKa 2.0 software^74^ with the PARSE^75^ force field and DelPhiPKa software^76^ with the AMBER force field.^77^ The p*K*_a_ values were calculated at physiological pH and ionic strength at 37 °C, following the instructions for each program. Two independent protocols were used for the evaluation of calculation accuracy.

## 3 Results

### 3.1 Octarepeat (OR) Region Exhibits a Mixture of Random Coil and PPII Helix

**Figure 2:**
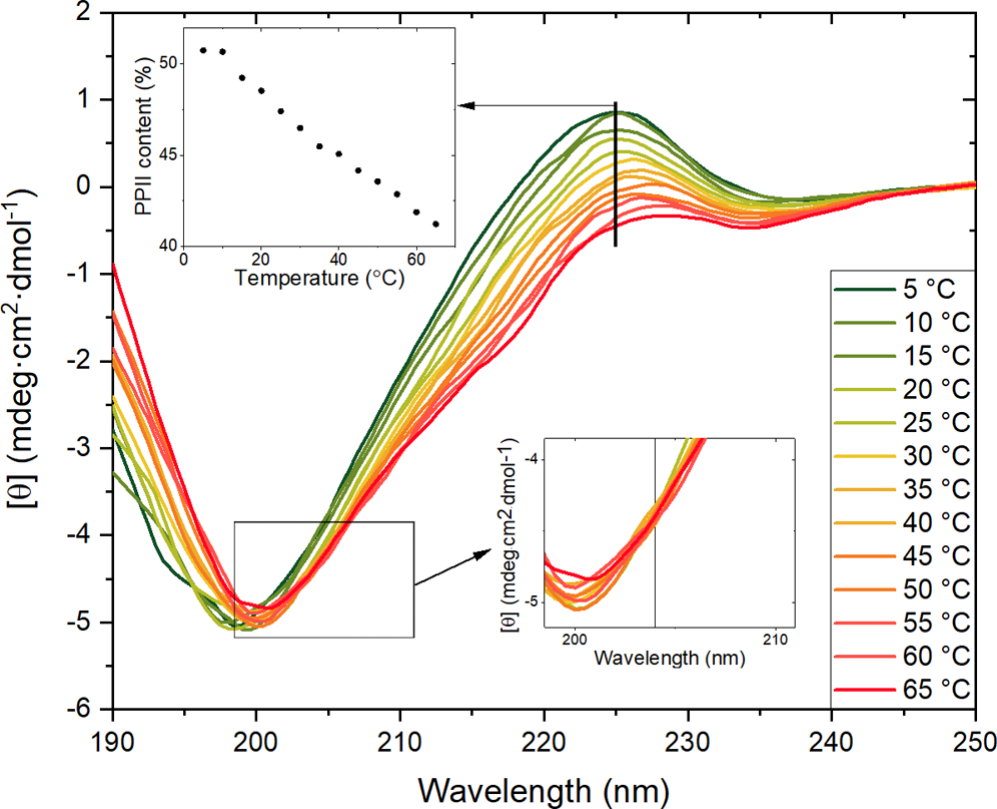
CD spectra showing the thermal unfolding of 20 μM OR peptide from 5 °C (dark green line) to 65 °C (dark red line) at 5 °C intervals. Bottom inset: the isodichroic point at 204 nm marked as a black vertical line for spectra from 20 to 65 °C (light green to red lines) suggests a PPII helix to random coil transition. Top inset: the estimated PPII helix content for all recorded temperatures, calculated from eq 1 and the CD intensities at 225 nm. All CD spectra were recorded in 10 mM phosphate buffer, pH 7.0.

The initial low-temperature (5 °C) CD spectrum of 20 μM *apo*-OR peptide showed one positive band at 225 nm, together with two negative bands: a weak one at 238 nm and a strong one at 199 nm (Figure 2, dark green spectrum). The minimum around 199 nm is consistent with a random coil structure, but this conformation should not give rise to positive CD bands, such as the one at 225 nm. Other researchers have previously suggested that the OR peptide in an aqueous solution adopts a PPII left-handed extended helix^78−80^ or exhibits a mixture of random coil and β-turn structures.^39,81,82^ To clarify the secondary structure of the OR peptide, we recorded CD spectra at different temperatures to monitor the thermal unfolding of the peptide (Figure 2).

For the temperatures in the range from 5 to 20 °C, the intensity of the CD spectra at 225 nm gradually decreased, but the spectral quality did not allow for any detailed interpretation of the spectral shape (Figure 2). For the spectra between 20 and 65 °C, however, an isodichroic point appeared at 204 nm (Figure 2, bottom inset, light green to red lines). Together with the gradual decrease of the 225 nm band, this indicates a structural transition from the PPII helix to random coil conformation.^83^ We therefore calculated the PPII content in the OR peptide as a function of temperature (Figure 2, top inset), using the CD signal intensity at 225 nm and eq 1.^59^ The PPII content was highest at 5 °C, i.e., around 51%, and then gradually decreased to around 41% at 65 °C. Interestingly, in the temperature range of the isodichroic point, i.e., 20–65 °C, the amount of PPII helix decreased linearly as a function of temperature. Overall, these CD results indicate that the secondary structure of the OR peptide at physiological temperature is a mixture of random coil and PPII helix, with a significant amount—more than 45%—of PPII structure.

### 3.2 Cu(II) and Zn(II) Binding to the OR Peptide both Induce Formation of an Antiparallel β-Sheet Structure

**Figure 3:**
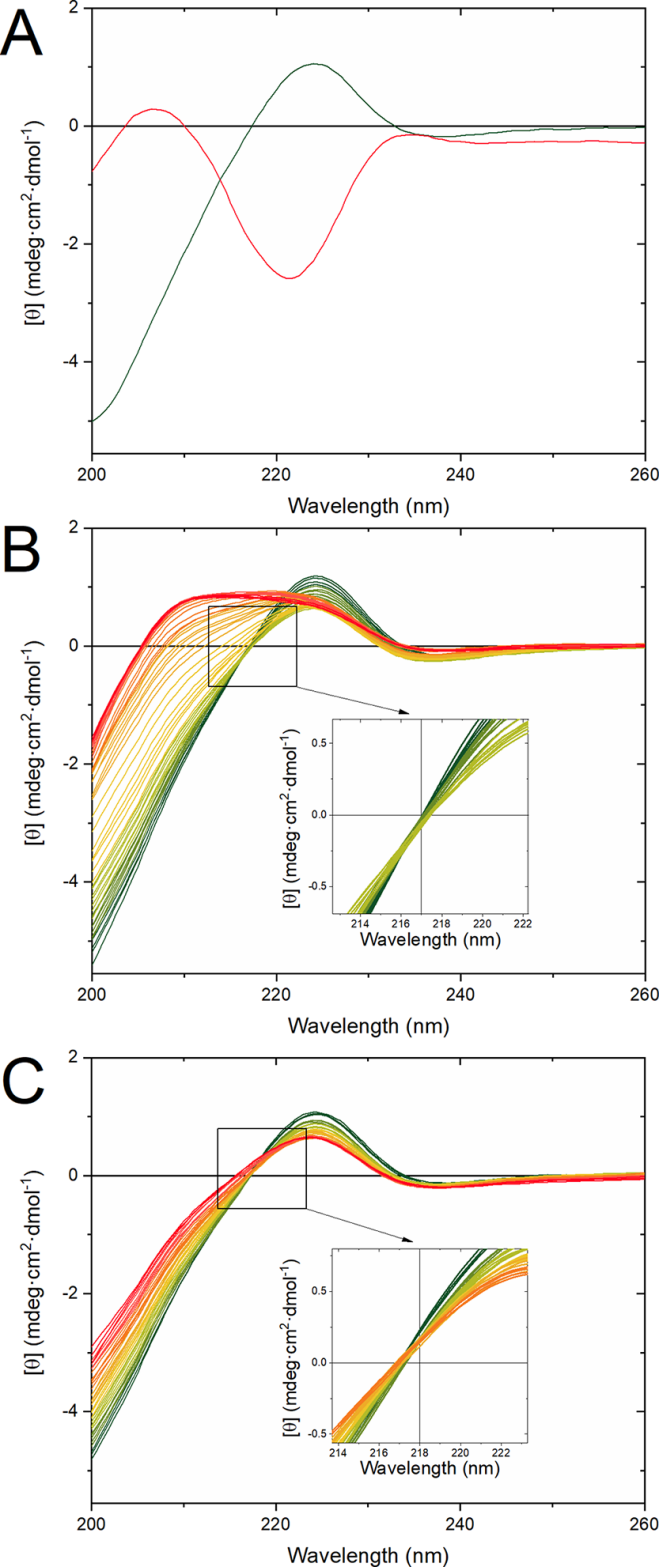
CD spectra showing titrations of Cu(II) and Zn(II) ions to the OR peptide at 25 °C. (A) Five micromolar OR peptide in water at pH 7.5 (no buffer) before (green line) and after (red line) addition of 20 μM CuCl_2_. The Cu(II) ions were added at pH 4.0, and then the pH was adjusted to 7.5 with small amounts of NaOH. The shape of the red CD spectrum shows features reported in the literature as typical for a Cu(II)–OR complex. (B) Titration of 5 μM OR peptide in 10 mM phosphate buffer, pH 7.5, with CuCl_2_ from 0 μM (green) to 40 μM (red). The color codes for the CuCl_2_ concentrations are shown in Figure S2. The inset shows the isodichroic point at 217 nm. (C) Titration of 5 μM OR peptide in 10 mM phosphate buffer, pH 7.5, with ZnCl_2_ from 0 μM (green) to 40 μM (red). The inset shows the isodichroic point close to 218 nm.

Previous studies suggest that Cu(II) binding to the OR peptide in pure water induces certain changes in the peptide’s secondary structure, involving the formation of β-turns or structured loops around the metal ions.^39^ Our initial titrations of 5 μM OR peptide with CuCl_2_ in water (pH adjusted to ∼7.5 with NaOH and controlled by a pH meter) showed a gradual decrease in the CD signal that is likely associated with peptide aggregation and precipitation (Figure S1A). The final spectrum had a weak single minimum at 220 nm and a maximum at 208 nm, which could suggest the formation of β-sheet structures. As the direct titrations with CuCl_2_ in water appeared to induce severe aggregation, an additional approach was tried. The 5 μM peptide solution was acidified with small amounts of acetic acid to pH ∼ 4.0. At pH 4.0, the histidine residues have hydrogen atoms on both the N^δ1^ and N^ε2^ nitrogen atoms, which makes them positively charged. They should therefore not bind positively charged Cu(II) ions, or at least they should bind them weaker. Next, 20 μM of CuCl_2_ was added, and the pH of the solution was gradually increased to ∼7.5 with small additions of NaOH. When the pH increases from acidic to neutral, the histidine residues undergo a transition from a charged state to a neutral state with only one hydrogen on either of the N^δ1^ or N^ε2^ nitrogen atoms. During this transition, the hydrogen-free N^δ1^ or N^ε2^ atom is available to bind a Cu(II) ion, and a gradual increase in pH, therefore, shifts the equilibrium toward the complex formation. This approach allowed us to acquire a CD spectrum with a shape that previously has been described in the literature as typical for the OR:Cu(II) complex and which has been interpreted as formation of β-turns or structured loops^39,80,81^ (Figure 3A, red spectrum). This spectrum was not caused by a pH effect (Figure S2).

To investigate the binding of Cu(II) ions to the OR peptide in a more controlled environment, we titrated CuCl_2_ to 5 μM OR peptide in 10 mM phosphate buffer, pH 7.5 at 25 °C (Figure 3B). The color codes for the CuCl_2_ concentrations are shown in Figure S3. The initial CD spectrum began to lose intensity at 224 nm, and a new band appeared at 208 nm. Careful analysis of these CD spectra revealed three distinct spectral transitions, likely corresponding to transitions in the peptide’s secondary structure. The first transition was present from 0 to 5 μM of CuCl_2_, corresponding to a 1:1 Cu(II)/OR peptide ratio. During this process, the CD intensity at 224 nm decreased, reaching a plateau, and a new weak band appeared at 208 nm (Figure 3B, green to yellow spectra). The isodichroic point at 217 nm was clearly visible for CuCl_2_ concentrations from 0 to 2 μM, which could suggest a PPII to β-turn structural transition.^84^ However, above 2 μM of CuCl_2_, the isodichroic point displayed a red shift, indicating a new, second transition.

The second spectral transition was remarkably visible at CuCl_2_ concentrations from 5 to 10 μM, corresponding to a 2:1 Cu(II)/OR peptide ratio. During this process, the CD intensity at 208 nm strongly increased, while the CD band at 224 nm showed a small intensity increase (Figure 3B, yellow to orange spectra). The absence of an observed isodichroic point excluded the possibility of a two-state transition, which suggests the formation of a new CD band and irreversible binding. The difference spectrum for this transition (Figure S4B, red line) resembled the CD spectrum for antiparallel β-sheets,^85^ and we therefore speculate that such β-sheet structures might have formed.

The third transition appeared for CuCl_2_ concentrations from 10 to 40 μM (Figure 3B, orange to red spectra). During this process, the newly formed band at 208 nm reached a maximum and maintained constant intensity, where the band at 224 nm began to lose intensity. No isodichroic point was observed, similar to the second transition, which again suggests the formation of a new spectral band and irreversible binding. The difference spectrum (Figure S4B, blue line) showed features similar to those observed in the second spectral transition, again suggesting the formation of an antiparallel β-sheet structure.^85^ However, as the changes in the CD spectral intensity were smaller for the third transition than for the second transition, less of the new structure appears to have formed.

The final CD spectrum for the OR·Cu(II) complex in the phosphate buffer has two maxima at 224 and 208 nm (Figure 3B, red spectrum), whereas the CD spectrum for the OR·Cu(II) complex in pure water has a maximum at 208 nm and a minimum at 220 nm (Figure 3A, red spectrum). Thus, the presence of the buffer may influence the structural transitions in the OR peptide during Cu(II) binding. The difference spectrum for the OR·Cu(II) complex in water has a maximum at 202 nm and a minimum at 222 nm (Figure S4A) and thus resembles the CD spectrum of a left-hand twisted antiparallel β-sheet, which is supposed to have a maximum at ∼203 nm and a minimum at ∼226 nm.^85^ This suggests that both in water and in phosphate buffer, Cu(II) binding to the OR peptide results in the formation of antiparallel β-sheet structures, however, possibly with different geometries.

Interestingly, titrating 5 μM OR peptide in the phosphate buffer with CuCl_2_ up to 40 μM, in 5 μM intervals, produced different CD spectra (Figure S1B) compared to the CuCl_2_ titrations with very small steps (Figure 3B). The final CD spectrum in the small-step titration had a maximum at 224 nm, and the formation of a new band at 208 nm was visible (Figure 3B, red spectrum). For the titrations with larger steps, i.e., 5 μM intervals, the isodichroic point at 217 nm was absent, the new band formed at 208 nm had lower intensity, and the band at 224 nm was not affected by the Cu(II) ions at all. The difference spectrum for titrations with 5 μM intervals (Figure S1B, blue line) shares similarities with the difference spectra for titrations with small steps (Figure S4B, red line, blue line) in the phosphate buffer, thus suggesting the formation of antiparallel β-sheet structures. We therefore suggest that during the large-step titrations, Cu(II) ions may have been bound in a rather chaotic way, possibly favoring intermolecular binding sites. In such a case, the Cu(II) binding would induce peptide aggregation, resulting in irreversible Cu(II) binding.

**Figure 4:**
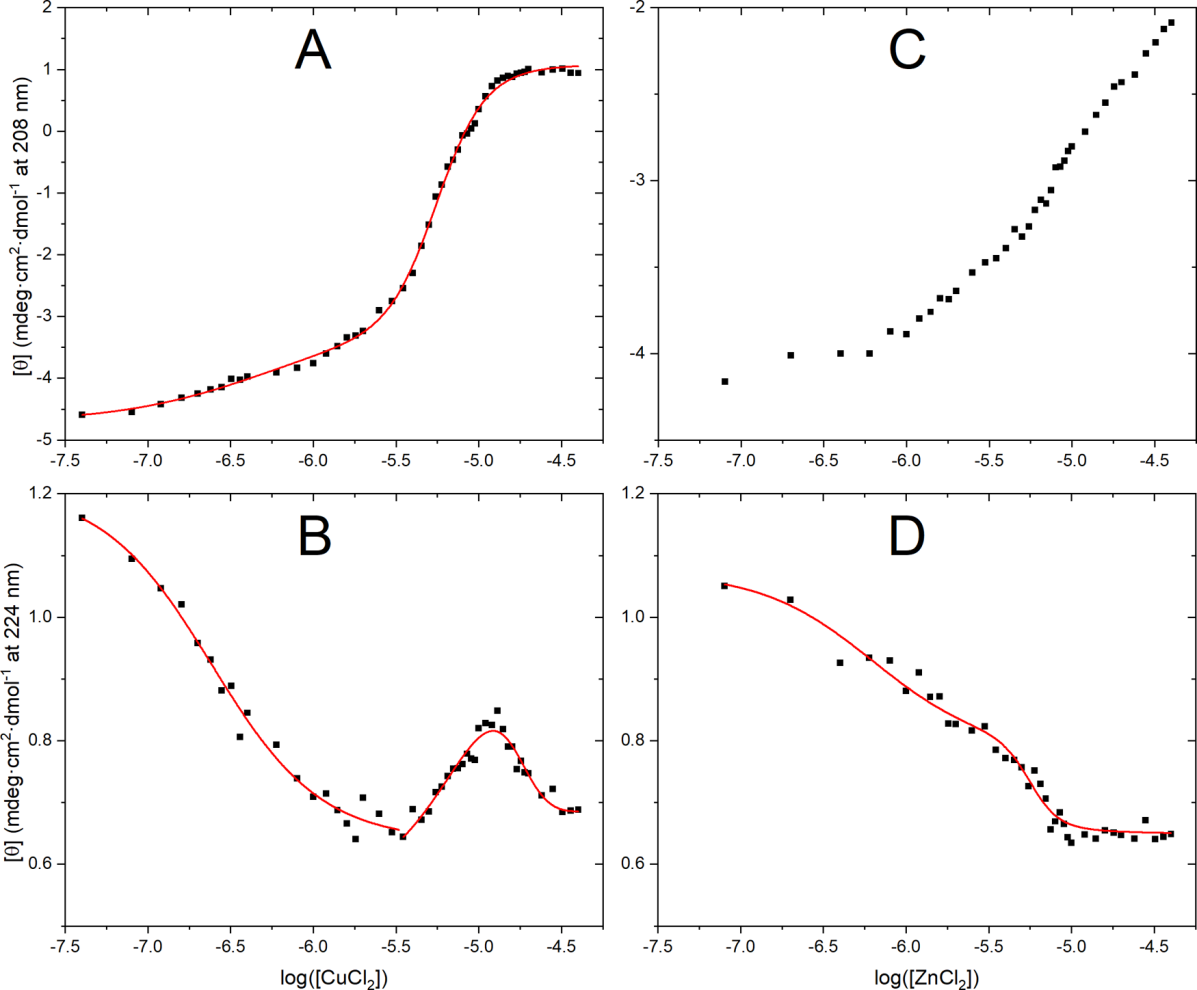
Changes in CD intensity [θ] for 5 μM OR peptide at 25 °C in 10 mM phosphate buffer, pH 7.5, when titrated with metal ions as shown in Figure 3. (A) 208 nm for Cu(II) ions (Figure 3B); (B) 224 nm for Cu(II) ions (Figure 3B); (C) 208 nm for Zn(II) ions (Figure 3C); and (D) 224 nm for Zn(II) ions (Figure 3C).

The titrations of the OR peptide with Cu(II) ions using small steps (Figure 3B) resembled a steady-state approximation; therefore, we analyzed these data in a more quantitative manner. Plots of the mean residue ellipticity at 208 and 224 nm versus CuCl_2_ concentration show all three spectral transitions in the OR peptide (Figure 4A,B). The data at 208 nm fitted with eq 3 yields apparent dissociation constants *K*_d1_^app^ of 0.52 μM for the first transition and *K*_d2_^app^ of 5.57 μM for the second transition. The change in CD intensity at 224 nm for the first transition fitted with eq 2 produced a *K*_d1_^app^ of 0.24 μM. The second and third transitions, visible at 224 nm and fitted to eq 4, produced the values *K*_d2_^app^ = 7.84 μM and *K*_d3_^app^ = 18.0 μM (Table 1). Thus, both the 208 and 224 nm CD data produced similar affinity values that were in the submicromolar range for the first transition and in the low micromolar range for the second transition. Moreover, the 208 and 224 nm CD data also produced similar Hill coefficients. The Hill coefficient for the first transition oscillates around 1. With an isodichroic point at 217 nm, most likely corresponding to a PPII helix to β-turn transition, the Hill coefficient of one suggests binding of a single Cu(II) ion. For the second transition, the Hill coefficient was between 2 and 3. Together with the lack of an isodichroic point, this suggests the formation of oligomeric forms such as dimers or trimers. For the third transition, the Hill coefficient was around 4. The lack of an isodichroic point for this transition suggests further formation of higher-order oligomeric species.

**Table 1:** Apparent Dissociation Constants (*K*_d_^app^) and Hill Coefficients (*n*_H_) for the OR-Metal Ion Complex, Based on Circular Dichroism (CD) and Fluorescence Quenching Experiments

metal ion	method	buffer	*K*_d1_^app^ [μM]	*n*_H1_	*K*_d2_^app^ [μM]	*n*_H2_	*K*_d3_^app^ [μM]	*n*_H3_
Cu(II)	CD at 208 nm	10 mM NaH_2_PO_4_, pH 7.5	0.52 ± 0.31	0.97 ± 0.48	5.57 ± 0.11	2.97 ± 0.25		
Cu(II)	CD at 224 nm	10 mM NaH_2_PO_4_, pH 7.5	0.24 ± 0.03	1.31 ± 0.20	7.84 ± 0.25	1.81 ± 0.14	18.0 ± 0.7	4.50 ± 0.41
Zn(II)	CD at 224 nm	10 mM NaH_2_PO_4_, pH 7.5	0.64 ± 0.17	1.28 ± 0.26	5.61 ± 0.37	5.11 ± 2.02		
Cu(II)	fluorescence at 355 nm	10 mM NaH_2_PO_4_, pH 7.5	4.29 ± 0.06	1.5 ± 0.03				
Cu(II)	fluorescence at 355 nm	10 mM MES, pH 7.5	4.5 ± 0.1	1.61 ± 0.05				

To investigate the Zn(II) binding to the OR peptide, we titrated 5 μM OR peptide with ZnCl_2_ in 10 mM phosphate buffer, pH 7.5, at 25 °C (Figure 3C). During the titrations with Zn(II) ions, the CD spectra of the OR peptide gradually lost intensity at 224 nm, and a weak new band appeared at 208 nm, similar to the CD titrations with Cu(II) ions. Plotting the CD signal intensities at 208 and 224 nm versus the ZnCl_2_ concentration showed two spectral transitions (Figure 4C,D).

The first transition appeared from 0 to 5 μM of ZnCl_2_ (Figure 3C, green to yellow spectra). During this process, the CD intensity at 224 nm decreased, and a new weak band at 208 nm appeared. A clear isodichroic point at 218 nm was visible, suggesting a reversible PPII to β-turn structural transition.^84^ The second transition was visible from 5 to 10 μM of ZnCl_2_ (Figure 3C, yellow to orange). During this process, the CD signal intensity at 224 and 208 nm decreased (Figure 4C,D), while the isodichroic point gradually shifted from 218 to 219 nm. The new isodichroic point suggests a two-state structural transition, possibly similar to the PPII to β-turn transition. The difference spectrum for this transition shows features similar to the first transition (Figure S4C, red and black lines). It may correspond to a reversible PPII transition into some other form of β-sheet structure. For ZnCl_2_ concentrations above 10 μM (Figure 3C, orange to red), no clear isodichroic point was observed, and the band at 208 nm gradually lost intensity, while the signal at 224 nm remained constant (Figure 4C,D). The difference CD spectrum for this process (Figure S4C, blue line) was similar to the difference spectrum for the OR peptide titrated with Cu(II) ions (Figure S4B, blue line), suggesting that further addition of Zn(II) ions also involves the formation of antiparallel β-sheet structures, although perhaps to a lesser degree.

As the CD intensities at 224 nm show two transitions, they can be fitted to eq 3 to produce apparent dissociation constants of *K*_d1_^app^ = 0.64 μM and *K*_d2_^app^ = 5.61 μM, for the first and second transition, respectively (Table 1). The Hill coefficient for the first transition is close to 1, which corresponds to the binding of a single Zn(II) ion. For the second transition, the Hill coefficient *n*_H2_ is 5.11 ± 2.02, and given the isodichroic point, it may correspond to a co-operative binding of a second Zn(II) ion. The CD intensity at 208 nm did not show a saturation or inflection point in the studied concentration range of ZnCl_2_ and was therefore not used for calculating binding affinity values.

Interestingly, the final CD spectrum for the Zn(II) titration, i.e., the OR peptide with 40 μM ZnCl_2_, is nearly identical to the CD spectrum for the OR peptide with 4 μM CuCl_2_ (Figure 3B,C). Characteristic features include: (i) decreased signal intensity at 224 nm and (ii) formation of a new band at 208 nm. But for the Cu(II) titration, this is just an intermediate step—the final step with 40 μM CuCl_2_ shows a very strong increase of the 208 nm band, which is something that Zn(II) ions apparently are not able to induce.

### 3.3 Protonation of His Residues Decreases the OR Peptide Affinity for Cu(II) and Zn(II) Ions

Tryptophan residues have previously been shown to indirectly coordinate Cu(II) ions in an HGGGW sequence, corresponding to the core of a single isolated octarepeat,^40^ and quenching of tryptophan fluorescence has been successfully applied to calculate the affinity between the octarepeat region and metal ions.^32,33,86,87^ Thus, we here used the intrinsic tryptophan fluorescence to further investigate the binding of Cu(II) and Zn(II) ions to the OR peptide. Cu(II) is a paramagnetic ion that can strongly quench nearby fluorophores. Zn(II) is diamagnetic, and any changes in tryptophan fluorescence upon added ZnCl_2_ should therefore originate only from zinc-induced changes in the peptide structure.

**Figure 5:**
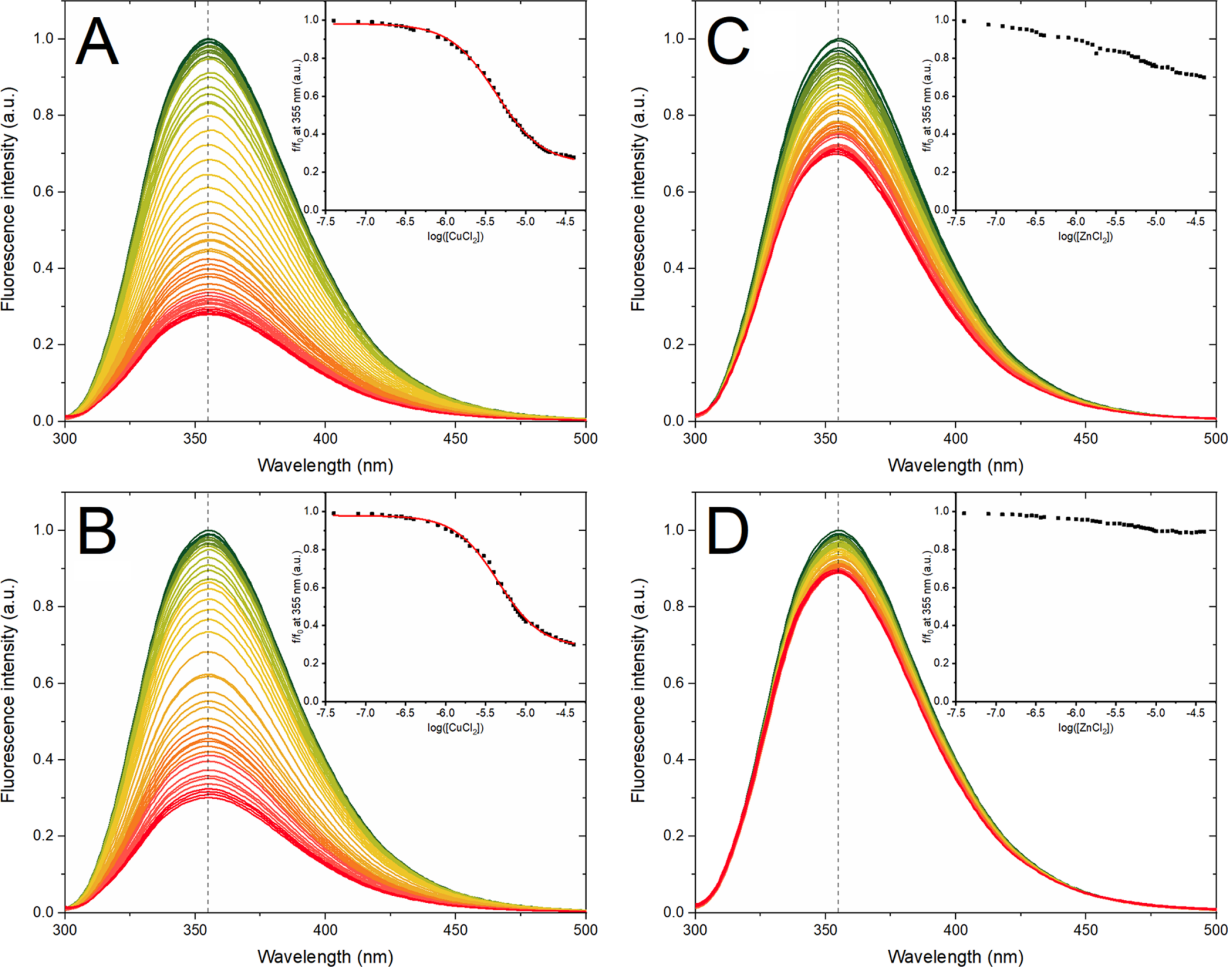
Fluorescence spectra for 5 μM OR peptide at 25 °C quenched with (A) CuCl_2_ in 10 mM phosphate buffer, pH 7.5; (B) CuCl_2_ in 10 mM MES buffer, pH 7.5; (C) ZnCl_2_ in 10 mM phosphate buffer, pH 7.5; and (D) ZnCl_2_ in 10 mM MES buffer, pH 7.5. The dashed vertical line at 355 nm shows no shift of the fluorescence maximum during the titration.

Cu(II) ions were titrated to 5 μM OR peptide at 25 °C in three sets of buffers: 10 mM phosphate buffer, pH 7.5; 10 mM MES buffer, pH 7.5, and 10 mM MES buffer, pH 5.5. As the OR peptide coordinates Cu(II) ions via histidine side chains,^40−42^ we expect the binding to be weaker at pH 5.5, where the His residues are protonated.^67^ In all three buffers, Cu(II) ions were found to quench the intrinsic tryptophan fluorescence, clearly demonstrating that Cu(II) ions bind to the OR peptide in all of the studied conditions. In all cases, the maximum fluorescence intensity was at 355 nm, and it did not change its position during the titrations. In buffers at pH 7.5, the Cu(II) ions quenched the tryptophan fluorescence to 30% of the initial intensity (Figure 5A,B), while in 10 mM MES buffer at pH 5.5, the tryptophan fluorescence intensity was reduced to 50% of the initial value (Figure S5A). To derive apparent *K*_d_ values, we plotted the fluorescence intensity at 355 nm as a function of the CuCl_2_ concentration and fitted the data to eq 2 (Figures 5A,B, and S5A insets). The calculated *K*_d_^app^ values were similar in the phosphate buffer and in the MES buffer at neutral pH, i.e., 4.3 and 4.5 μM, respectively (Table 1). At acidic conditions, the signal did not saturate in the studied CuCl_2_ concentrations, suggesting a weaker binding. The *K*_d_^app^ values derived with fluorescence spectroscopy are close to those derived for the second Cu(II) ion with CD spectroscopy, although it should be pointed out that different buffers were used (Table 1). MES is a “Good” buffer with minimal metal binding,^88^ but it is not suitable for CD measurements. Phosphate buffer is compatible with most spectroscopic techniques and biologically relevant, but the phosphate ions have some weak interactions with metal ions.

To investigate if the OR peptide also binds Cu(I) ions, we performed tryptophan fluorescence titrations with CuCl_2_ under reducing conditions obtained with 1 mM TCEP. Copper predominantly occurs as Cu(II) extracellularly and as Cu(I) intracellularly due to the reducing environment of the cytosol.^89^ It is therefore important to clarify how the two different types of copper ions interact with the OR region. Our results showed that titrating Cu(I) ions to 5 μM OR peptide in 10 mM MES buffer, pH 7.5, with 1 mM TCEP, clearly quenched the tryptophan fluorescence (Figure S5C), demonstrating binding of Cu(I) to the OR peptide. As the fluorescence signal did not saturate with the studied Cu(I) concentrations, it appears that the OR peptide has a weaker affinity for Cu(I) ions than for Cu(II) ions, and the *K*_d_ for the Cu(I) ions is probably above 40 μM.

Titrations with Zn(II) ions to 5 μM OR peptide at 25 °C were performed in the same three buffers as the titrations with CuCl_2_, i.e., 10 mM phosphate buffer, pH 7.5; 10 mM MES buffer, pH 7.5; and 10 mM MES buffer, pH 5.5. The changes in the OR tryptophan fluorescence at pH 7.5 were stronger in the phosphate buffer than in the MES buffer, indicating a buffer effect on the zinc binding (Figure 5C,D). At pH 5.5, no significant changes in fluorescence intensity were observed (Figure S5B), indicating that the OR peptide does not bind Zn(II) ions at acidic conditions. In all titrations, the fluorescence maximum remained positioned at 355 nm. The weaker fluorescence quenching by Zn(II) ions compared to Cu(II) ions is to be expected, as Zn(II) ions are not paramagnetic. However, the observed quenching effect does show that also Zn(II) ions bind to the peptide at neutral pH, even though the signal-to-noise ratio is too low to quantitatively evaluate the binding curves.

### 3.4 Binding of Cu(II) or Zn(II) Ions to the OR Peptide Induces Formation of Hairpin Structures

**Figure 6:**
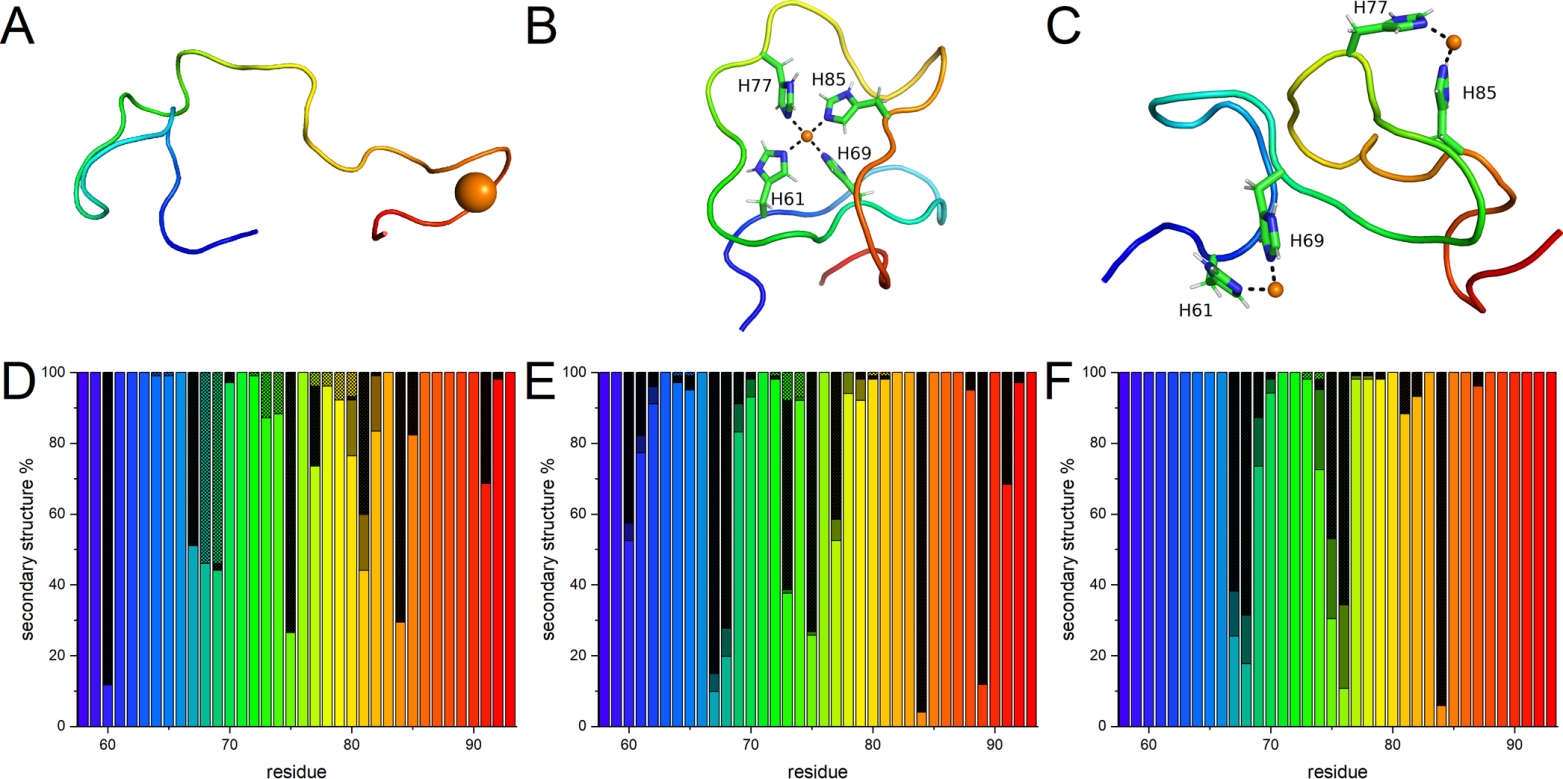
Endpoint snapshots and secondary structure distributions of the OR peptide simulated with (A, D) a single Cu(II) ion and protonated histidine N^ε2^ atoms; (B, E) a single Cu(II) ion and neutral histidine N^ε2^ atoms; and (C, F) two Cu(II) ions and neutral histidine N^ε2^ atoms. The secondary structures were determined for each generated model using the PROSS^72^ algorithm: β-turns are marked with checker filling, polyproline II helices with black, β-strands with gray, and coils have no filling.

**Figure 7:**
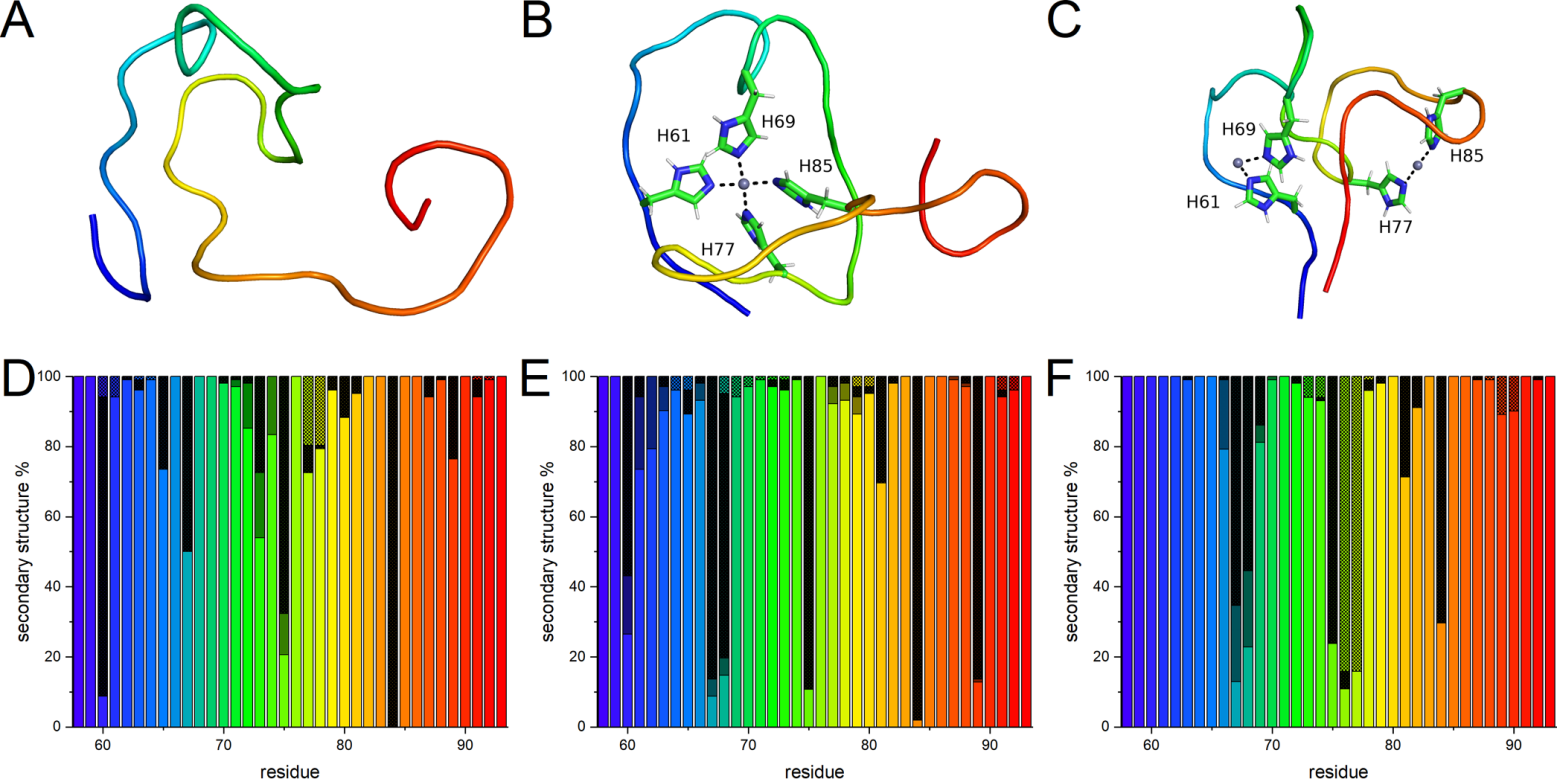
Endpoint snapshots and secondary structure distributions of the OR peptide simulated with (A, D) a single Zn(II) ion and protonated histidine N^ε2^ atoms; (B, E) a single Zn(II) ion and neutral histidine N^ε2^ atoms; and (C, F) two Zn(II) ions and neutral histidine N^ε2^ atoms. The secondary structure distributions were calculated using the PROSS method: β-turns are marked with checker filling, polyproline II helices with black, β-strands with gray, and coils have no filling.

**Figure 8:**
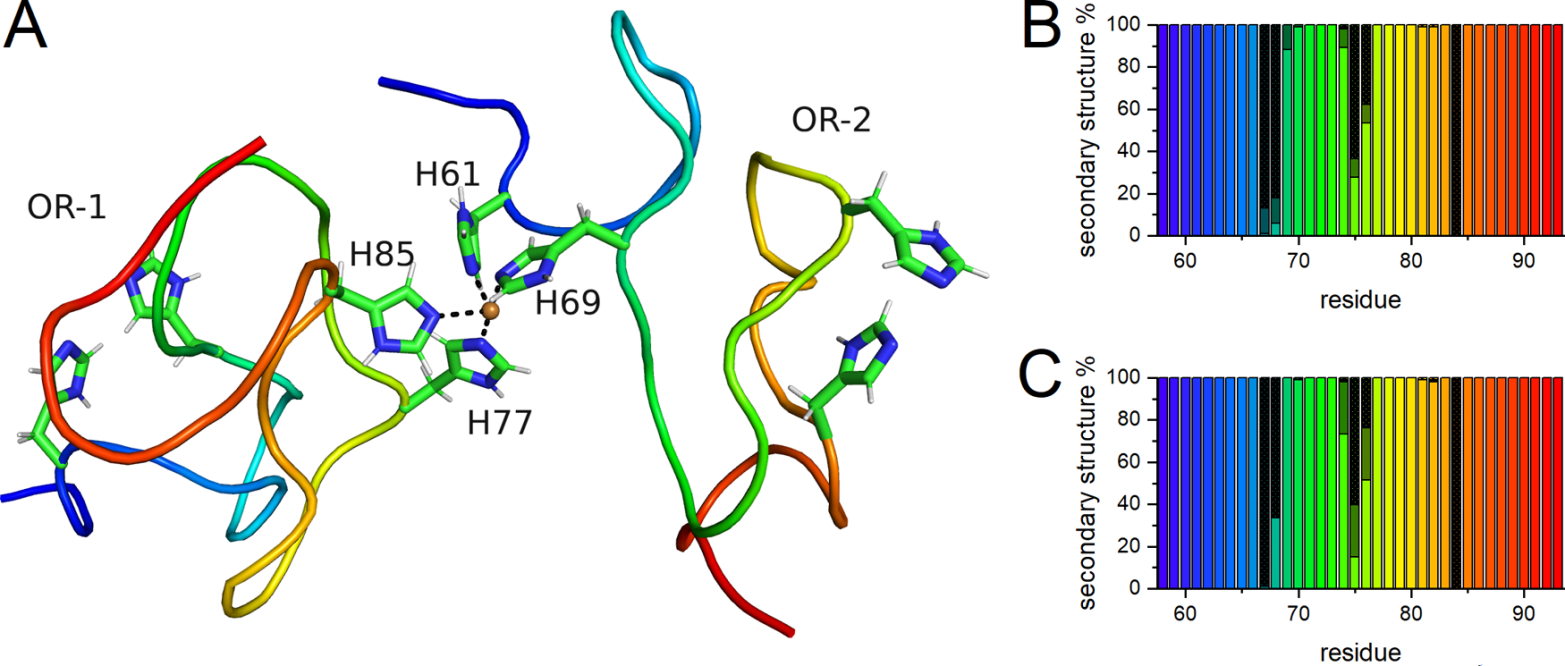
Endpoint snapshot (A) of two OR peptide molecules simulated with a single bound Cu(II) ion. The N-terminus and C-terminus of the first OR peptide (OR-1) are marked in blue and green, and the N-terminus and C-terminus of the second OR peptide (OR-2) are marked in green and red, respectively. The secondary structure distributions for the two OR peptides are shown in (B) (OR-1) and (C) (OR-2) and were calculated using the PROSS method: β-turns are marked with checker filling, polyproline II helices with black, and coils have no filling.

Molecular dynamics (MD) simulations were carried out to characterize the structural transitions in the OR peptide, i.e., PrP^C^(58–93), when bound to Cu(II) or Zn(II) ions. Final models from the MD simulations are shown in Figures 6–8, and visualizations of the first principal components of the simulations are shown in Figure S6.

To study interactions corresponding to acidic pH, a single Cu(II) ion was positioned next to four protonated histidine N^ε2^ atoms. As expected, the Cu(II) ion rapidly moved away from the protonated His residues during the MD equilibration phase. The simulations quickly converged, as shown by the root-mean-square deviation (RMSD) values (Figure S7A). During the whole simulation time, the Cu(II) ion remained bound to the Cβ main chain carbonyl groups of residues His85, Gly87, Gly88, and Trp89, suggesting nonspecific electrostatic binding (Figure 6A). The OR peptide in this protonation state adopted an extended and flexible conformation with a root-mean-square fluctuation (RMSF) around 0.5 nm (Figure S8A), where the secondary structure predominantly consisted of coils with some regions showing propensities for β-turn and PPII helix conformations (Figure 6D). Principal component analysis (PCA) suggested that the main conformational changes involved motions in the N- and C-termini (Figure S6A).

Next, we simulated the OR peptide with either one or two Cu(II) ions located next to the neutral N^ε2^ atoms of the four OR histidine residues, corresponding to the OR peptide at neutral pH. Both simulations quickly converged (Figure S7A). The single Cu(II) ion remained bound by all four histidine residues and two axially bound water molecules over the whole simulation time (Figure S9A). In this binding mode, the peptide backbone formed multiple loops around the Cu(II) ion, while residues His61–Gly71 formed a hairpin-like structure stabilized by Cu(II)-bound His61 and His69 (Figure 6B). This model had smaller RMSF values than the OR peptide model with protonated histidine residues (Figure S8A), indicating a more rigid structure. Indeed, the main PCA component showed smaller backbone displacements near the Cu(II) ion than at the N- and C-termini (Figure S6B). Moreover, in this binding mode, more OR residues adopted the PPII helix and transient β-strand secondary structures compared to the OR peptide with protonated histidine residues (Figure 6E). For the simulations with two Cu(II) ions, each copper ion remained bound by two histidine residues and four water molecules (Figure 6C) over the whole simulation time (Figure S9B). In this binding mode, the peptide backbone formed a structure with three hairpins. The first hairpin was stabilized by one of the Cu(II) ions and was located near the N-terminus, involving residues Gly62–His69. The second hairpin, which was stabilized by the other Cu(II) ion, involved residues Gly74–His85 and was thus located near the C-terminus. The third hairpin involved residues His69–Pro76 and formed a bridge between the two Cu(II)-bound segments (Figure 6C). The RMSF data showed intermediate values, with minima corresponding to the histidine residues involved in Cu(II)-binding (Fig. S8A). The primary PCA component suggests that the two Cu(II)-stabilized hairpin structures were rigid in themselves but could move relative to each other, resulting in the formation of the middle bridging hairpin (Figure S6C). Secondary structure analysis indicated a reduction of the PPII structure and formation of antiparallel β-strands around residues Gln67–Gly70 and Gly74–Pro76, i.e., roughly the region of the middle bridging hairpin (Figure 6F).

Similar simulations were also performed for the OR peptide with Zn(II) ions. For the OR peptide with fully protonated histidine residues and simulated with a single Zn(II) ion, the metal ion moved away from the peptide in the MD equilibration phase and remained unbound for the whole simulation time. The peptide adopted an elongated (Figure 7A) and flexible conformation with an average RMSF of 0.6 nm (Figure S8B). The first PCA component suggests that the main movement in the OR peptide involves motions in the N- and C-termini (Figure S6D). During the simulation time, the OR peptide mainly adopted coil and PPII secondary structures (Figure 7D).

In the next step, we simulated the OR peptide with neutral N^ε2^ atoms, together with one or two Zn(II) ions. Both simulations converged (Figure S7B). The single Zn(II) ion placed next to the four histidine residues remained bound over the whole simulation time (Figure S9C) and was additionally coordinated by two axially bound water molecules. In this binding mode, the peptide backbone again formed multiple loops around the Zn(II) ion, and residues Gly74–Gly86 formed a hairpin structure stabilized by the zinc ion bound to His77 and His85. The average value of the Cα RMSF was 0.3 nm, which is a much smaller value than for the OR peptide with protonated histidine residues (Figure S8B). The first PCA component suggests that the main movement of the peptide backbone occurred in the hairpin loop (residues Gly78–Gly82) and the C-terminal loop (residues 86–91), while the backbone around the Zn(II) ion had smaller mobility (Figure S6E). Analysis of the secondary structure showed that more residues formed a β-strand secondary structure than for the OR peptide with protonated histidine residues (Figure 7E). However, the peptide mainly adopted coil and PPII helix secondary structures.

When two Zn(II) ions were positioned close to the histidine residues, both ions remained bound over the whole simulation time, and each one was coordinated by two histidine residues and four water molecules (Figure S9D). In this binding mode, the peptide backbone formed four hairpin structures. Two hairpin structures involved residues His61–His69 and His77–His85, respectively, and each one of them was stabilized by a single Zn(II) ion. Then, two bridging hairpins involving residues Gln67–Trp73 and Gln75–Gly78 were located between the two Zn(II)-stabilized hairpins (Figure 7C). The peptide backbone had an average RMSF of 0.4 nm (Figure S8B), indicating higher Cα fluctuations than when only one Zn(II) ion was bound to the OR peptide. The primary PCA component suggests that in this binding mode, the main motion of the peptide backbone corresponds to the formation of the two bridging hairpin structures (i.e., residues Gln67–Trp73 and Gln75–Gly78) and end-to-end interactions involving the terminal residues Gly58, Gln59 and Gly92, Gly93 (Figure S6F). Analysis of the peptide’s secondary structure showed a reduced PPII helix content but an increase in the coil and β-turn secondary structures when two Zn(II) ions were bound (Figure 7F).

In the last step, we simulated two OR peptide molecules with neutral N^ε2^ atoms bound to a single Cu(II) ion. The initial model contained the Cu(II) ion bound to His77 and His85 from the first OR molecule (OR-1) and to His61 and His69 from the second OR molecule (OR-2). The initial models of OR-1 and OR-2 were taken from the final step of our MD simulation of OR with two bound Cu(II) ions (Figure 6C). The simulation converged and surprisingly showed a minimal RMSD over time (Figure S7A). The average value of the Cα RMSF was below 0.05 nm (Figure S8C), which is the smallest value from all simulations performed in this study. The single Cu(II) ion placed next to the four histidine residues remained bound during the entire simulation time (Figure S9E). The three hairpin structures, involving residues Gly62–His69, His69–Pro76, and Gly74–His85, were preserved in each OR molecule (Figure 8A). Secondary structure analysis indicated similar structural compositions for the OR-1 and OR-2 molecules. Both peptide molecules predominantly adapted coil structures, with a PPII structure at residues Gln67, Pro68, Gln75, Pro76, and Pro84 in OR-1 and OR-2, and β-strands at residues Gln67–His69, Gly74–Pro76 in OR-1, and Gly74–Pro76 in OR-2 (Figure 8B,C).

### 3.5 Calculation of p*K*_a_ Values for the OR Peptide Histidine Residues

Protein binding affinities for Cu(II) and Zn(II) ions can be affected by the p*K*_a_ values of the His residue side chains, which are known to be close to the physiological pH.^67,90^ Changes in p*K*_a_ values can be observed in structural conformations that favor hydrogen bond interactions with the histidine side chains, and such p*K*_a_ changes can affect the binding affinity for metal ions.

We therefore calculated p*K*_a_ values for side chains of histidine residues from different final MD conformations (Table 2). To increase the accuracy of the calculations, two independent protocols were used, namely, PropKa 2.0 and DelPhiPKa.^74,76^ The two protocols gave p*K*_a_ values for all four His residues that are within ±6% of the average calculated values (Table 2).

**Table 2:** p*K*_a_ Values for the Four Histidine Residues in the OR Peptide, Calculated for the Final MD Models from Simulations with a Single Bound Metal Ion and either Protonated (Figures 6A and 7A) or Neutral Histidine Residues (Figures 6B and 7B), and with either the PropKa 2.0 or DelPhiPKa 2.3 Protocol

	final MD models from simulations with neutral histidine residues		final MD models from simulations with protonated histidine residues	
	Cu(II)	Zn(II)	Cu(II)	Zn(II)
PropKa 2.0				
His61	5.87	6.88	6.50	6.43
His69	6.41	6.01	6.50	6.43
His77	6.20	6.11	6.43	6.29
His85	6.48	7.15	6.47	6.50
DelPhiPKa 2.3				
His61	5.64	5.88	6.43	6.43
His69	6.11	6.06	6.45	6.43
His77	6.10	6.12	6.49	6.29
His85	6.16	6.18	6.46	6.50

The two protocols consistently show that both the position of the His residue and the protein conformation have noticeable effects on the calculated p*K*_a_ value (Table 2). For the final models from the MD simulations, both protocols produce slightly higher p*K*_a_ values for His85 than for the other three residues. His85 is the histidine most exposed to the solvent, and ionic interactions with water molecules are energetically more favorable than hydrogen bonds. For the same reasons, both protocols consistently show slightly higher p*K*_a_ values for the final models from simulations with charged His residues than for models from simulations with neutral His residues.

## 4 Discussion

### 4.1 Solution Structure of the OR Peptide

Previous studies have suggested that the OR peptide in an aqueous solution adopts a combination of random coil structure together with either PPII left-handed extended helix^78−80^ or β-turn structures.^39,81,82^ Our CD results for the apo-OR peptide, i.e., PrP^C^(58–93), show unfolding at elevated temperatures (Figure 2), thus demonstrating the existence of secondary structures different from the random coil at low temperatures. In the 20–65 °C temperature range, an isodichroic point at 204 nm indicates a PPII helix to random coil transition.^84^ This is consistent with previous studies of the OR peptide,^78−80^ and also similar to our earlier results on structural transitions in the amyloid-β (Aβ) peptides involved in Alzheimer’s disease.^83^ Temperature studies of the Aβ(1–40) peptide and its shorter N-terminal fragments generally show an isodichroic point around 208 nm, a weak positive band at ∼222 nm that becomes negative at high temperatures, and a strong negative band at ∼200 nm whose intensity is reduced at high temperatures. The similar results obtained here for the OR peptide suggest similar structures and temperature-induced structural transitions in the two peptides. Our results suggest that ∼45% of the OR peptide is in PPII conformation at 37 °C, with the remaining structure being random coils. For the Aβ(1–40) peptide, the corresponding numbers are ∼30% PPII helix and ∼70% random coil structure.^83^ The lack of a well-defined isodichroic point below 20 °C (Figure 2) indicates that the OR peptide can form various secondary structures at low temperatures. This is consistent with NMR studies of the OR sequence at 20 °C, which suggest the presence of structured loops as well as β-turns.^91^

### 4.2 Metal Binding to the OR Peptide Induce Formation of β-Sheet Secondary Structure

Earlier studies have shown that the OR region can bind up to four Cu(II) ions, but no detailed structural model for the OR peptide backbone during Cu(II) binding has been proposed. More than 20 years ago, Viles et al. performed far-UV CD titrations of the OR peptide with Cu(II) ions in water at pH 7.4.^39^ The addition of Cu(II) ions was found to decrease the intensity of both a negative band at 200 nm and a positive band at 225 nm and to induce a new negative band at 222 nm together with a new positive band at 204 nm. This result was interpreted as a structural alteration corresponding to the formation of β-turns or structured loops.^39^ Later studies have suggested that the negative band at 222 nm might reflect the structures of tryptophan side chains.^30^ In this study, we were able to largely recreate the results of Viles et al., but we present a different interpretation. The difference spectrum for the Cu(II)–OR complex in water (Figure S4A) has a strong negative band at 222 nm and a strong positive band at 202 nm. It thereby resembles the CD spectrum for left-handed twisted antiparallel β-sheets,^85^ as well as the CD spectrum for a hydrophobic fragment of the Aβ peptide, i.e., Aβ(25–35), to which we previously have attributed an antiparallel β-sheet secondary structure.^83^ We therefore suggest that binding of Cu(II) ions to the OR peptide in water results in the formation of antiparallel β-sheet structures.

We further propose that antiparallel β-sheet structures are also formed when Cu(II) ions bind to 5 μM OR peptide in the phosphate buffer, pH 7.5, but these β-sheet structures appear to have a different geometry. The addition of up to 5 μM CuCl_2_, corresponding to a 1:1 OR/Cu(II) molar ratio, produces an isodichroic point at 217 nm, which suggests a two-state PPII helix to β-turn reversible transition. Above 5 μM of CuCl_2_, the lack of an isodichroic point suggests irreversible binding, and the appearance of a new CD band at 208 nm may be caused by the formation of some types of antiparallel β-sheet structures.^85^ The intensity increase for the new 208 nm band was higher for Cu(II) concentrations in the 5–10 μM range than for the 10–40 μM interval, suggesting that binding of two Cu(II) ions to the OR peptide is a primary event responsible for β-sheet formation. Therefore, further binding of two additional Cu(II) ions, up to a total of four Cu(II) ions, increases the formation of antiparallel β-sheet structures, however, to a lesser amount.

A CD spectrum from an earlier study, i.e., of 50 μM of Syrian hamster OR peptide together with 250 μM of Cu(II) ions in 20 mM ammonium acetate at pH 6.0, has been interpreted as representing a Cu(II)–OR complex.^92^ The CD spectrum of that study^92^ is almost identical to our CD spectrum for 5 μM human OR peptide with 10 μM of Cu(II) ions in 10 mM phosphate buffer, pH 7.5 (Figure 3B, orange lines). The CD spectrum of the Syrian hamster OR–Cu(II) complex was, however, different from the previously reported human OR–Cu(II) complex in water at pH 7.4,^39^ which is consistent with our current observations (Figure 3A,B), and which confirms that the buffer conditions influence the structure of the complex.

In another study, no changes in the CD spectrum were reported when Cu(II) ions were titrated to the human OR peptide in 10 mM phosphate buffer, pH 7.0.^80^ That observation was interpreted as the OR peptide being unable to bind Cu(II) ions in phosphate buffer due to competition with Cu(II)–phosphate binding. Although the OR peptide concentration was not specified in that study, and the CD data were shown only for one CuCl_2_ concentration, the reported CD spectra look like they can have an isodichroic point around ∼220 nm, which is close to our observed isodichroic point at 217 nm (Figure 3B). In living organisms, Cu(II) ions rarely exist in free form,^36^ and phosphate ions readily form complexes with Cu(II) ions.^93^ However, during our titrations, we have not observed any precipitation of copper phosphate. If the binding of Cu(II) ions to phosphate was so much stronger than binding to the OR peptide, then Cu(II) binding to the OR peptide would be physiologically irrelevant, given the high concentration of free phosphate ions in the extracellular fluid.^94^ Our current data clearly show that the OR peptide binds both Cu(II) and Zn(II) ions in various buffers, including phosphate buffer.

As the OR region is known to bind multiple—up to four—Cu(II) ions, the binding conformations depend on the Cu(II) concentration.^41,42,81,86^ The first Cu(II) ion is coordinated by four N^ε2^ atoms from the four histidine residues.^41,42^ This binding mode has the highest affinity, with a *K*_d_ below 3 nM.^32,42^ Binding of the second Cu(II) ion rearranges the binding site, and each Cu(II) ion becomes coordinated by two histidine sidechain N^ε2^ atoms,^41^ possibly in combination with negatively charged atoms, and the *K*_d_ is ∼200 nM.^42^ Further addition of up to four Cu(II) ions rearranges the binding configuration so that each Cu(II) ion is coordinated by one histidine sidechain N^δ1^ atom and three negatively charged atoms, such as two deprotonated amide nitrogen atoms and one carbonyl oxygen atom from the preceding glycine residues.^40−42^ In this binding mode, the OR peptide has the weakest affinity for Cu(II) ions, with a *K*_d_ in the 1–10 μM range.^32,42^ However, it should be stated that in our experiments, we can only measure apparent *K*_d_ values, which are higher than the real *K*_d_ values. Measurements of the real binding affinities would require peptide concentrations ten times smaller than the expected *K*_d_ value, which in this case is below the detection limit of CD instruments.

The OR peptide backbone is expected to adopt different conformations for each binding configuration. Our CD titrations confirm this notion (Figure 3B), and the transitions are particularly clear at 224 nm (Figure 4B). Up to 5 μM CuCl_2_, i.e., 1:1 Cu(II)/OR ratio, the intensity at 224 nm monotonically decreased with added Cu(II) ions. From 5 to 10 μM CuCl_2_, i.e., 1:1 to 2:1 Cu(II)/OR ratio, the intensity at 224 nm monotonically increased. From 10 to 40 μM CuCl_2_, i.e., 2:1 to 8:1 Cu(II)/OR ratio, the intensity at 224 nm decreased and reached a plateau around ∼20 μM CuCl_2_, i.e., around 4:1 Cu(II)/OR ratio. These observations are clearly consistent with the binding of one, two, and up to four Cu(II) ions to the OR peptide in the three intervals of CuCl_2_ concentration (Figure 4B).

The binding of Zn(II) ions to the OR peptide in 10 mM phosphate buffer, pH 7.5, seems to induce similar secondary structures in the OR peptide as Cu(II) ions. Again, the changes in CD intensity at 224 nm seem to reflect distinct structural transitions (Figure 3C). For concentrations of up to 5 μM of ZnCl_2_, i.e., 1:1 Zn(II)/OR ratio, the 224 nm intensity monotonically decreased with added Zn(II) ions, suggesting binding of a single Zn(II) ion in this interval (Figure 4D). The isodichroic point at 218 nm suggests that the binding of the first Zn(II) ion is reversible. From 5 to 10 μM of ZnCl_2_, i.e., from 1:1 to 2:1 Zn(II)/OR ratios, the 224 nm intensity decreased even more steeply, suggesting the binding of two Zn(II) ions in this interval. The isodichroic point at 219 nm suggests that also the binding of the second Zn(II) ion is reversible. Above 10 μM of added ZnCl_2_, no further changes appear in the CD spectrum at 224 nm, indicating that the binding of two Zn(II) ions saturates the OR peptide. Other researchers have suggested that all four OR histidine residues are involved in binding a single Zn(II) ion.^51,52,95^ This might be true when the OR peptide binds Zn(II) ions in a 1:1 molar ratio, but at higher Zn(II) concentrations, we suggest that two Zn(II) ions are bound, each one by two histidine residues, in a manner similar to that for two Cu(II) ions.

Surprisingly, the outcome of the metal ion binding measurements depends on the size of the titration steps. The addition of Cu(II) ions in 5 μM steps produced a new CD band without any isodichroic point (Figure S1B), which suggests irreversible binding. In our previous studies, we have shown that immediately after the addition of 40 μM Zn(II) ions to 10 μM OR peptide, the peptide was able to bind amyloid-specific dyes like Thioflavin T and Congo Red, and after longer incubation times, it formed amyloid fibrils with the cross-β structure,^54^ which is an irreversible process. In contrast, the addition of Zn(II) ions in small steps produced what appears to be a reversible process. This suggests that in biological systems of metal release, such as the synaptic cleft, not only the total amount but also the rate of metal release may contribute to amyloid formation.

### 4.3 Histidine Protonation and Metal Ion Binding to the OR Peptide

Our fluorescence spectra of the OR peptide titrated with Cu(II) ions at pH 7.5 suggest an apparent *K*_d_ ∼ 4.5 μM (Table 1). This value is close to the mean apparent dissociation constant of 7.2 μM previously reported for an isolated HGGGW repeat.^32^ Calculating real *K*_d_ values requires competition experiments with, e.g., glycine.^32^ As our main objective was to show changes in metal ion binding affinity under different conditions, no competition experiments were performed in this study. Our CD experiments for the OR peptide titrated with Cu(II) ions clearly show three spectral transitions, which we attribute to the binding of four Cu(II) ions. The single apparent *K*_d_ value from our fluorescence experiments therefore seems to represent the average *K*_d_ from all three possible Cu(II) binding modes.^42^ Our fluorescence titrations under acidic conditions (pH 5.5) show that the OR peptide can bind Cu(II) ions also under acidic conditions where the histidine residues are protonated,^67^ however, with a lower affinity.

The histidine residues′ p*K*_a_ values were found to be between 6 and 7, depending on the degree of solvent exposure and the hydrogen bonds created (Table 2). Earlier studies with mass spectrometry indicate that the OR peptide can bind only up to two Cu(II) ions at pH 6.0, i.e., less than the four Cu(II) ions that can be bound at pH 7.4.^92^ Thus, our fluorescence quenching measurements at pH 5.5 (Figure S5A) may reflect binding of two Cu(II) ions to the OR peptide.

Experiments involving a reducing agent, i.e., 1 mM TCEP, which roughly corresponds to the intercellular reducing environment, showed only a weak tryptophan quenching effect by Cu(I) ions, and the signal did not saturate during the experiments. It therefore appears that the OR region mainly binds Cu(II) ions.

Tryptophan fluorescence measurements of the OR peptide titrated with ZnCl_2_ at pH 7.5 showed reduced fluorescence intensity indicative of metal binding, but the poor signal-to-noise ratio of the data did not allow calculation of the binding affinity (Figure 5C,D). At pH 5.5, the addition of ZnCl_2_ did not affect the intrinsic OR peptide fluorescence at all (Figure S5B), indicating that the OR peptide does not bind Zn(II) ions under acidic conditions.

### 4.4 Models for Formation of β-Sheet Secondary Structure

The molecular dynamics simulations of the OR peptide with metal ions generally support our CD results. The secondary structure of the OR peptide with protonated histidine residues, in the presence of a single Cu(II) or Zn(II) ion, mainly consists of the coil structure, although some PPII helix structures (12% overall) are present around residues Pro60, Gln67, Gln75, and Pro84 (Figures 6D and 7D). The amount of PPII helix in the MD simulations is much smaller than calculated from our CD spectra, probably due to the imperfection of the applied OPLS-AA force field to recreate the PPII structure.^96^ When the OR peptide with neutral histidine residues is modeled together with a single bound metal ion, the peptide again mainly formed a coil structure, although together with four transient β-strands. With a bound Cu(II) ion, the transient β-strands were formed by Pro60–Gly62, Gln67–Gly70, Trp73–Gln75, and His77–Gly79. With a bound Zn(II) ion, the transient β-strands were Pro60–Gly63, Gly66–Pro68, His77–Gln79, and Gly87–Trp89. Thus, most of the β-strands appeared in close proximity to the histidine residues involved in metal ion binding, i.e., His61, His69, His77, and His85. The overall amount of PPII helix structure was around 15% in these simulations, with the main contributions coming from Pro60, Gln67, Pro68, Gln75, Pro84, and Trp89.

Although the OR peptide simulated with neutral histidine residues and two bound metal ions mainly adopted a coil structure, we observed significant reductions in the PPII helix structure (9% overall), which is in line with our CD analysis. The PPII helix content was distributed here in a more uniform way, mainly around the Pro68, Pro76, and Pro84 residues. Furthermore, modeling with two bound Cu(II) ions (Figure 6C) induced the formation of only two β-strands consisting of residues Gln67–Gly70 and Gly74–Pro76, i.e., mainly in the middle of the bridging hairpin structure (residues His69–Pro76). For two bound Zn(II) ions, a similar β-strand was formed at Gly66–Gly70, together with two accompanying short β-turns at Trp73–Gly74 and Pro76–His77. In this case, the β-structures formed were located around the first (residues Gln67–Trp73) and second (residues Gln75–Gly78) bridging hairpins, which connect the N-terminal and C-terminal hairpin structures stabilized by the two metal ions (Figure 7C). These observations are consistent with our CD results, where the formation of β-turn secondary structure was observed for the second spectral transition (i.e., for binding of the second Zn(II) ion (Figure 4D)).

Structural models with a single bound metal ion from our MD simulations agree with previously published results for a single Cu(II) ion bound to the OR peptide, where the metal-binding site remained exposed to the solvent and the peptide backbone formed multiple loops around the Cu(II) ion.^97,98^ Interestingly, Pushie et al. performed molecular dynamics simulations for two octapeptide repeats, each bound with a single bound Cu(II) ion.^99^ The authors observed stacking of the two HGG metal-binding regions on each other, where the GWGQ linker region formed a bend or a turn structure. Those models resemble our hairpin structures from the simulation of the OR peptide with two Cu(II) ions. In the “stacked” models, two copper binding sites were located in close proximity, with a copper–copper distance of ∼5 Å. With two neighboring histidine imidazole moieties, it is possible that the 2:1 binding mode (two Cu(II) ions to one OR peptide) under higher Cu(II) occupancy undergoes rearrangement to a 4:1 binding mode, with the hairpin properties being preserved, which would agree with our CD results. Future modeling with multiple OR peptides (or full-length PrP^C^) and multiple metal ions may shed more light on the structural effects of metal binding to aggregated peptides/proteins.

### 4.5 OR Peptide Metal Ion Binding and Amyloid Formation

The OR region is an important part of the PrP^C^ molecule, likely being involved in the protein’s neuroprotective effect.^100^ Previous studies have suggested that Cu(II) and Zn(II) ions may inhibit the in vitro conversion of PrP^C^ to PrP^Sc^ by the formation of nonamyloid aggregates.^101^ On the other hand, we have previously reported that after the addition of 4 molar equiv of Zn(II) ions, the OR peptide forms structured fibrils that display the cross-β structure typical for amyloid material and which bind the amyloid-specific dyes Thioflavin T and Congo Red.^54^ Thus, the OR peptide incubated with an excess of Cu(II) ions may also form amyloid aggregates. Our CD titrations and MD simulations of the OR peptide bound with two metal ions (i.e., either two Cu(II) or two Zn(II) ions) showed the formation of β-structures, which is consistent with our previous results. In our MD simulations, the OR peptide formed β-structures in the Gln67–Gly70 and Gly74–Pro76 regions. As β-structures—especially hairpins—are favorable for amyloid formation,^18^ we speculate that these regions may form a core for amyloid aggregation of the OR peptide. However, our CD and MD results indicate that binding of a single metal ion to the OR peptide induces only minor changes in the peptide’s secondary structure. Thus, it is possible that only high concentrations of metal ions—more than a 1:1 ratio—would induce secondary structures suitable for amyloid formation. In such cases, the TSE diseases might be a result of altered Cu(II) or Zn(II) homeostasis.^25,26,53^ On the other hand, the binding of divalent metal ions to full-length PrP^C^ is known to induce interactions between the metal-bound OR region and the C-terminal helical domain.^28,95,102^ Such interactions likely help to hold the protein structure together, and it is generally known that structured proteins must first unfold before they can misfold into β-sheet structures (or β-sheet hairpins) and begin to assemble into amyloid forms. In a PrP^C^ variant with point mutations corresponding to genetic Creutzfeldt–Jakob disease, fatal familial insomnia, and the Gerstmann–Sträussler–Scheinker disease, the addition of Zn(II) ions induced broadening of NMR peaks indicative of weaker interactions between the Zn(II)-bound OR region and the C-terminal domain^95^ than in the native protein. Thus, the metal-induced interactions between the OR region and the C-terminal domain likely counteract amyloid formation in the full-length protein by stabilizing the protein fold, and these stabilizing interactions may be weaker in some disease-related PrP^C^ mutants. Finally, metal ions may promote aggregation by binding to the OR region of two or more PrP^C^ proteins, thereby bringing the two proteins together, which is a first step toward aggregation. Our MD simulations (Figure 8) showed that such intermolecular Cu(II) coordination was stable over time, indicating that complexes with Cu(II) ions and two or more PrP^C^ molecules are likely to form in vivo.

The relation between metal binding and PrP^C^ aggregation is clearly complex, with two effects that likely promote aggregation (one metal ion binding to multiple PrP^C^ molecules and bound metal ion inducing β-sheet structures suitable for amyloid formation) and one effect that likely counteracts amyloid formation (stabilizing the protein fold by interactions between the C-terminal domain and the metal-bound OR region). Our tentative understanding is that the effects that promote aggregation will dominate at high concentrations of metal ions, i.e., at metal/protein ratios higher than 1:1. As four Cu(II) ions but only two Zn(II) ions could bind to one OR peptide, and as Cu(II) ions had a larger effect on the peptide structure than the Zn(II) ions, amyloid formation is probably more efficiently induced by Cu(II) than by Zn(II) ions.

## 5 Conclusions

In summary, our results show that the OR region in the PrP^C^ protein can bind up to four Cu(II) ions or two Zn(II) ions. The first metal ion binds with a submicromolar apparent dissociation constant, and further metal ions bind with low micromolar apparent dissociation constants (Table 1). The OR histidine residues are important binding ligands, where Zn(II) binding is more sensitive to histidine protonation than Cu(II) binding. The metal ions can be coordinated by histidine residues from different PrP^C^ molecules—such intermolecular complexes appear to be stable first steps towards protein aggregation. Without bound metal ions, the secondary structure of the OR peptide is a combination of random coil and PPII helix. The addition of metal ions induces structural changes into β-sheet conformations, which generally are beneficial for amyloid aggregation. The structural conversions are most prominent for large concentrations (i.e., above 1:1 ratio) of Cu(II) ions, suggesting that especially Cu(II) ions could be an important factor in converting the PrP^C^ protein into amyloids of the neurotoxic PrP^Sc^ form.^58^

## Supporting Information Available

CD spectra of the OR peptide titrated with CuCl_2_; effect of pH on the OR peptide CD spectrum; color codes for spectra from figures; difference CD spectra; fluorescence spectra for the OR peptide quenched with CuCl2 and ZnCl2; the first component of the principal component analysis; root-mean-square deviation of the OR peptide atomic position during MD simulations; root-mean-square fluctuations of OR peptide Cα atoms during MD simulations; and distances between metal ions and histidine residues in function of time (PDF)

## Accession Codes

The UniProt ID number for the human prion protein is P04156.
