# Supplementary material for: Prion Protein Octarepeat Domain Forms Transient β-Sheet Structures upon Residue-Specific Binding to Cu(II) and Zn(II) Ions
Source: Biochemistry. 2023 May 10;62(11):1689–705. doi: 10.1021/acs.biochem.3c00129 (PMC10249355; doi:10.1021/acs.biochem.3c00129)
Supplement: Supplementary file 1 — bi3c00129_si_001.pdf [file bi3c00129_si_001.pdf]

Supporting information

The Prion Protein Octarepeat Domain Forms Transient β-sheet Structures Upon Residue-Specific Binding to Cu(II) and Zn(II) Ions

Maciej Gielnik [1, 2], Aneta Szymańska [3], Xiaolin Dong[4], Jüri Jarvet [4,5], Željko M. Svedružić [6], Astrid Gräslund [4], Maciej Kozak [1,7], and Sebastian K. T. S. Wärmländer [4,*]

1 Department of Macromolecular Physics, Faculty of Physics, Adam Mickiewicz University, PL 61-614 Poznań, Poland; mkozak@amu.edu.pl (M.K)

2 Department of Molecular Biology and Genetics, Aarhus University, DK 8000 Aarhus, Denmark; maciejgielnik@mbg.au.dk (M.G.)

3 Department of Biomedical Chemistry, Faculty of Chemistry, Gdańsk University, PL 80-308 Gdańsk, Poland; aneta.szymanska@ug.edu.pl (A.S.)

4 Chemistry Section, Stockholm University, 10691 Stockholm, Sweden; seb@student.su.se (S.K.T.S.W.); jyri.jarvet@dbb.su.se (J.J.); astrid@dbb.su.se (A.G.)

5 The National Institute of Chemical Physics and Biophysics, 12618 Tallinn, Estonia

6 Department of Biotechnology, University of Rijeka, HR 51000, Rijeka, Croatia; zeljko.svedruzic@biotech.uniri.hr (Ž.S.)

7 National Synchrotron Radiation Centre SOLARIS, Jagiellonian University, PL 30-392 Kraków, Poland

* Corresponding author.

Fig S1: CD spectra of the OR peptide titrated with $CuCl_2$ S2
Fig S2: Effect of pH on the OR peptide CD spectrum S2
Fig S3: Color codes for CD titrations curves in Figure 3 S3
Fig S4: Difference spectra for CD curves in Figure 3 S4
Fig S5: Fluorescence spectra for the OR peptide titrated at acidic pH or with reducing agent TCEP S4
Fig S6: Visualization of the first principal component of OR peptide simulations S5
Fig S7: Root mean square deviation (RMSD) for OR peptide simulations S5
Fig S8: Root mean square fluctuations (RMSF) for simulations of OR peptide Cα atoms S6
Fig S9: Distances between metal ions and histidine Nε2 atoms in OR peptide simulations S6

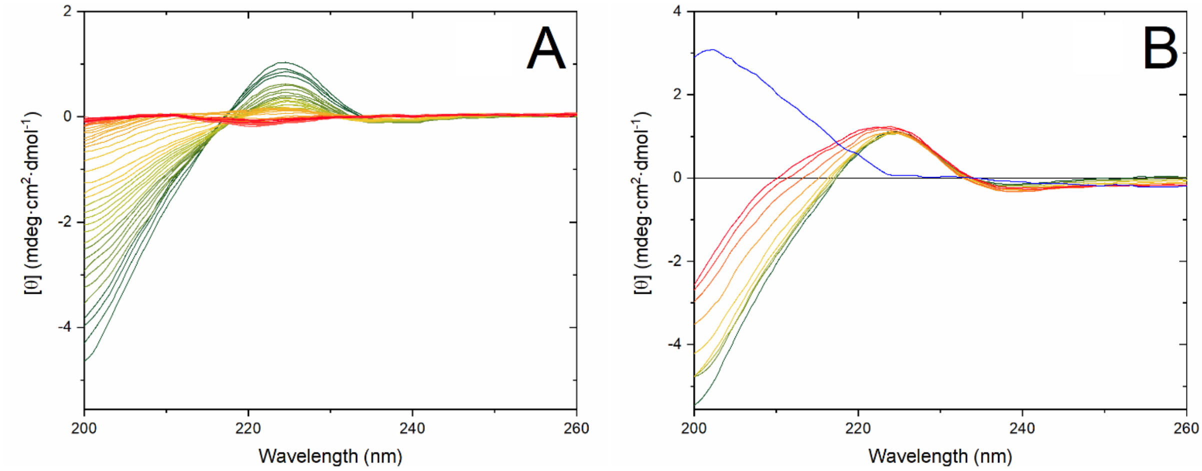

Figure S1. CD spectra of the OR peptide titrated with $CuCl_2$. (**a**) Titration of 5 µM OR peptide in pure water at 25 °C, with $CuCl_2$ in increasing (0.04 µM, 0.2 µM, 0.5 µM, 2 µM and 4 µM) intervals. The pH was adjusted to 7.5 with small amounts of NaOH. Initial spectrum: blue. Final spectrum (40 µM $CuCl_2$): red. (**b**) Titration of 5 µM OR peptide in 10 mM phosphate buffer, pH 7.5, with $CuCl_2$ in 5 µM intervals. Initial spectrum: green. Final spectrum (40 µM $CuCl_2$): red. Difference spectrum between the initial and final states: blue.

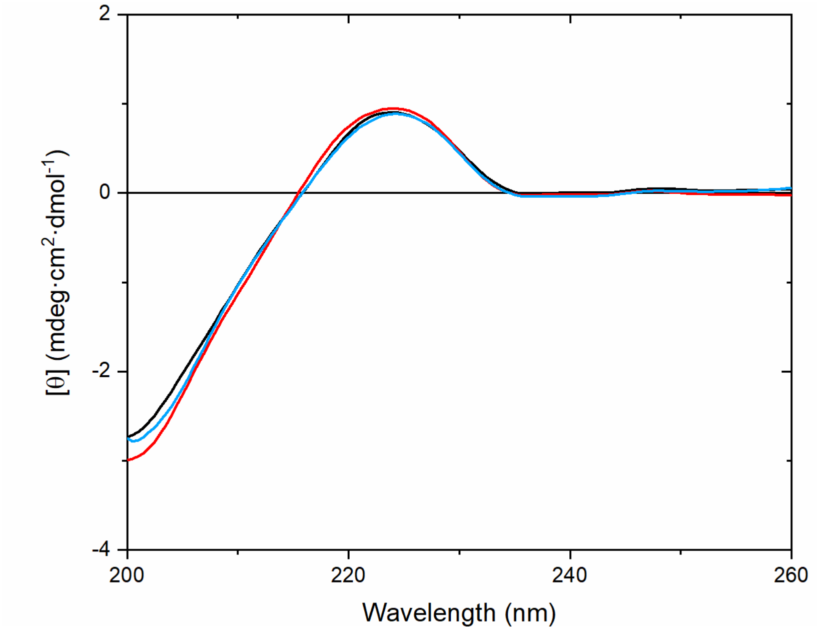

Figure S2. Effect of pH on OR peptide CD spectrum. 5 µM OR peptide dissolved in water with the pH adjusted to 4.0 (red), 5.5 (black) or 7.5 (blue) with small amounts of acetic acid or sodium hydroxide.

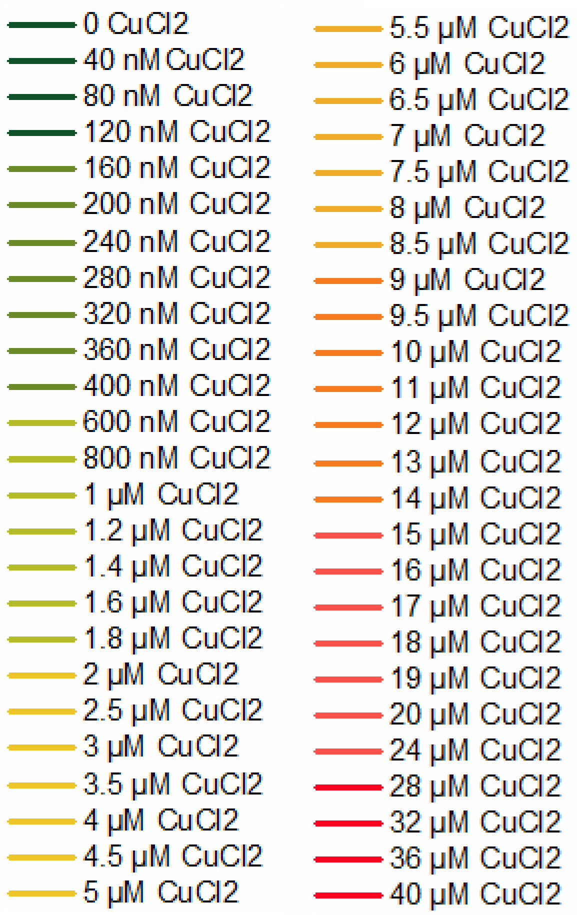

Figure S3. Color codes for spectra from Fig. 3B. Similar color codes were applied for $ZnCl_2$ in Fig. 3C and Fig. S1.

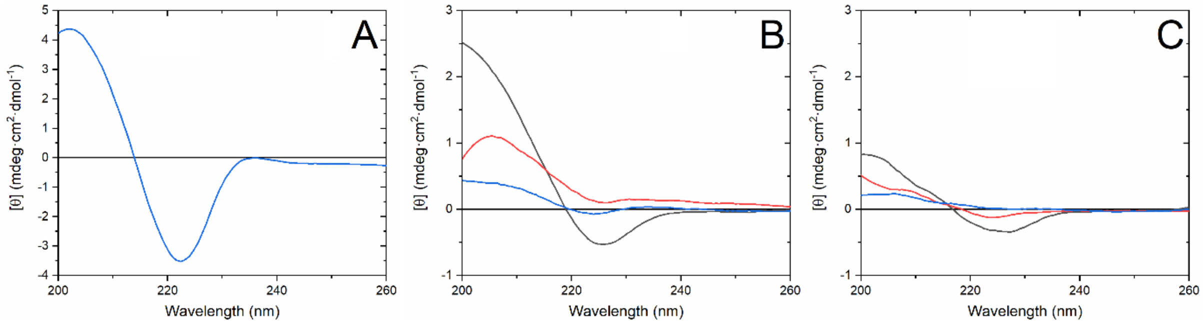

Figure S4. Difference spectra for CD curves in Figure 3. (**a**) Difference between the two spectra shown in Fig. 3a, i.e., 5 µM OR peptide in water at pH 7.5 before and addition of 20 µM $CuCl_2$. (**b**) Difference spectra between the first and last spectrum of the first (black - 5 µM OR peptide with 5 µM $CuCl_2$ minus 5 µM OR peptide), second (red - 5 µM OR peptide with 10 µM $CuCl_2$ minus 5 µM OR peptide with 5 µM $CuCl_2$), and third (blue - 5 µM OR peptide with 20 µM $CuCl_2$ minus 5 µM OR peptide with 10 µM $CuCl_2$) transitions shown in Figure 3b; (**c**) Difference spectra between the first and last spectrum of the first (black - 5 µM OR peptide with 5 µM $ZnCl_2$ minus 5 µM OR peptide), second (red - 5 µM OR peptide with 10 µM $ZnCl_2$ minus 5 µM OR peptide with 5 µM $ZnCl_2$), and third (blue - 5 µM OR peptide with 20 µM $ZnCl_2$ minus 5 µM OR peptide with 10 µM $ZnCl_2$) transitions shown in Figure 3c.

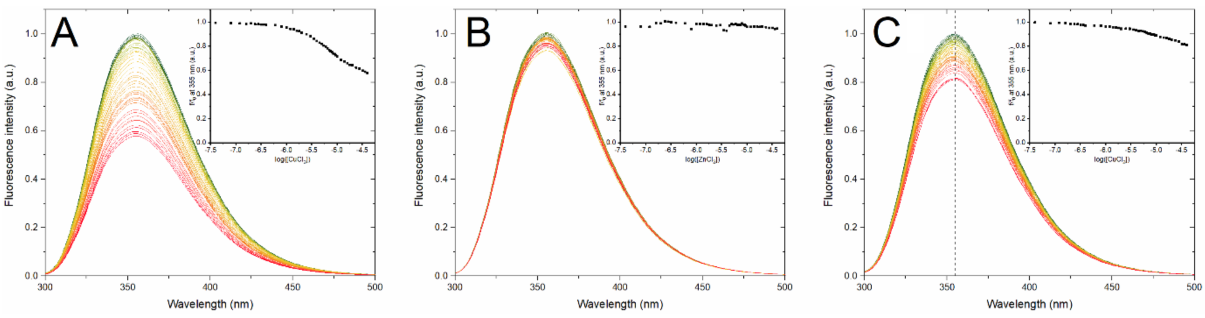

Figure S5. Fluorescence spectra for 5 µM OR peptide at 25 °C quenched with: (**A**) $CuCl_2$ in 10 mM MES buffer, pH 5.5; (**B**) $ZnCl_2$ in 10 mM MES buffer, pH 5.5; (**C**) $CuCl_2$ in 1 mM TCEP, 10 mM MES buffer, pH 7.5.

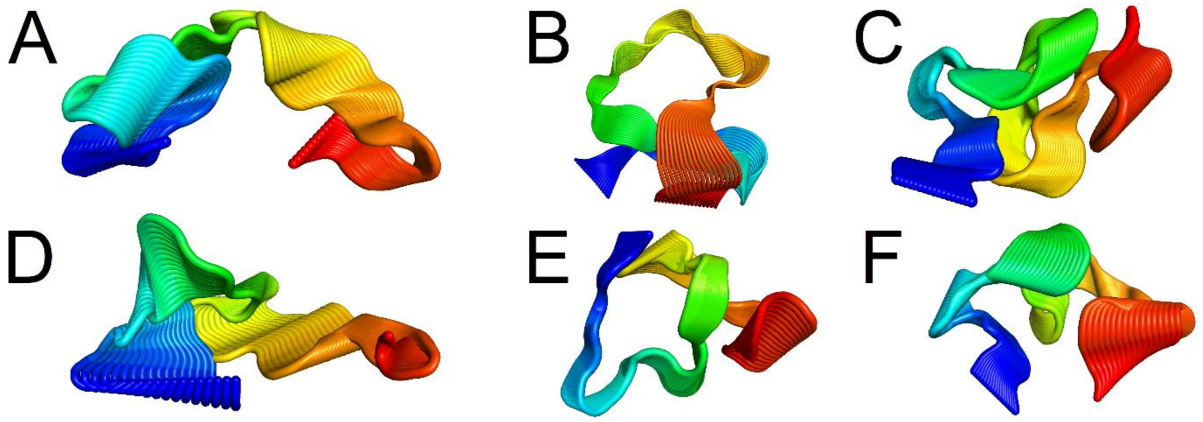

Figure S6. Visualization of the first principal component of the OR peptide simulated together with (**a**) a single Cu(II) ion and protonated histidine residues, (**b**) a single Cu(II) ion bound to four $N^{\varepsilon 2}$ atoms of neutral histidine residues, (**c**) two Cu(II) ions, each bound to two $N^{\varepsilon 2}$ atoms of neutral histidine residues, (**d**) a single Zn(II) ion and protonated histidine residues, (**e**) a single Zn(II) ion bound to four $N^{\varepsilon 2}$ atoms of neutral histidine residues and (**f**) two Zn(II) ions, each bound to two $N^{\varepsilon 2}$ atoms of neutral histidine residues.

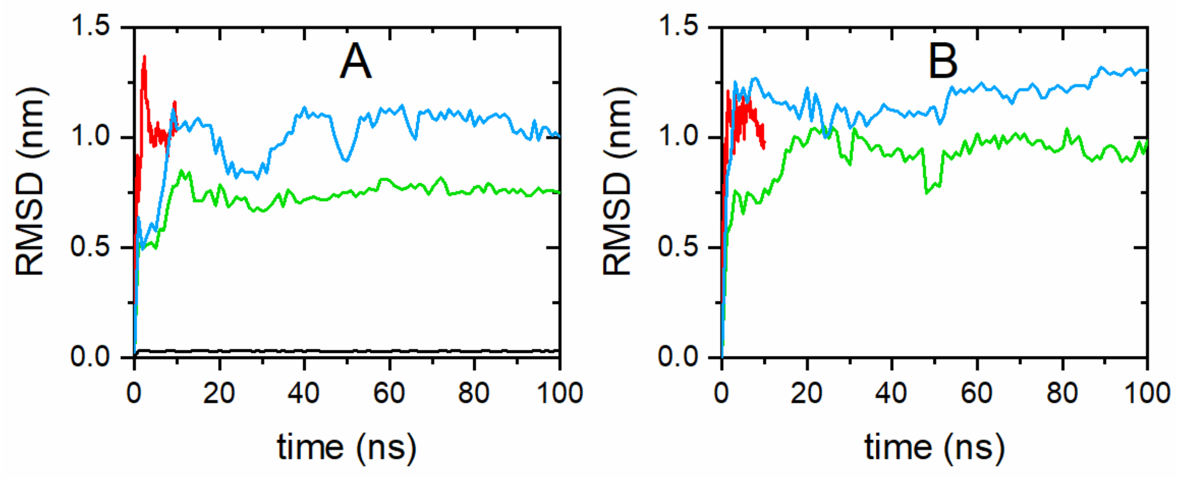

Figure S7. Root mean square deviation (RMSD) of atomic positions for simulations of the OR peptide together with: (**a**) Cu(II) ions, and (**b**) Zn(II) ions. The OR peptide with fully protonated histidine residues and a single metal ion (i.e., either Cu(II) or Zn(II)) is shown in red, while the OR peptide with neutral histidine residues simulated with a single metal ion is shown in green, the OR peptide with neutral histidine residues simulated with two metal ions is shown in blue, and the two OR peptides with neutral histidine residues simulated with a single metal ion are shown in black.

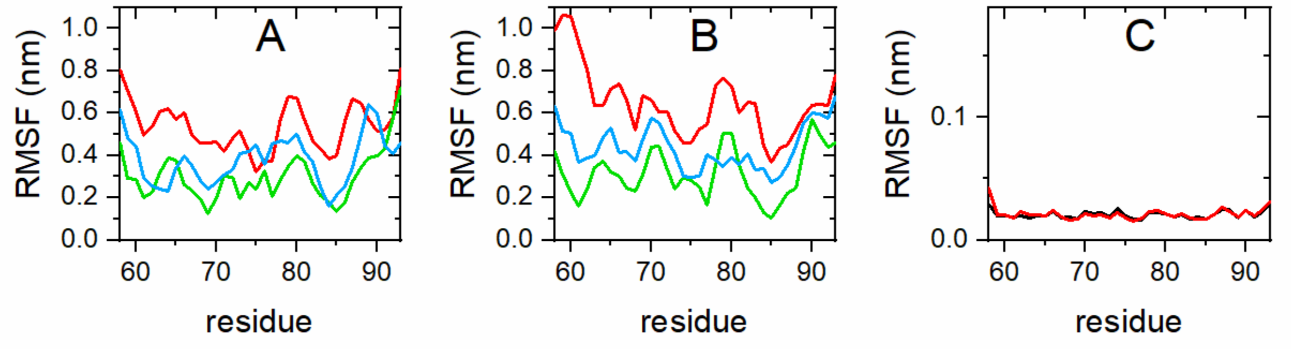

Figure S8. Root mean square fluctuations (RMSF) for simulations of the OR peptide Cα atoms together with: (**a**) Cu(II) ions, and (**b**) Zn(II) ions. Simulations of the OR peptide with fully protonated histidine residues and a single metal ion (i.e., either Cu(II) or Zn(II)) are shown in red, while the OR peptide with neutral histidine residues and a single metal ion are shown in green, and the OR peptide with neutral histidine residues and two metal ions are shown in blue. RMSF for (**c**) two OR peptide molecules simulated with neutral histidine residues and a single Cu(II) ion. OR-1 data is black and OR-2 data red.

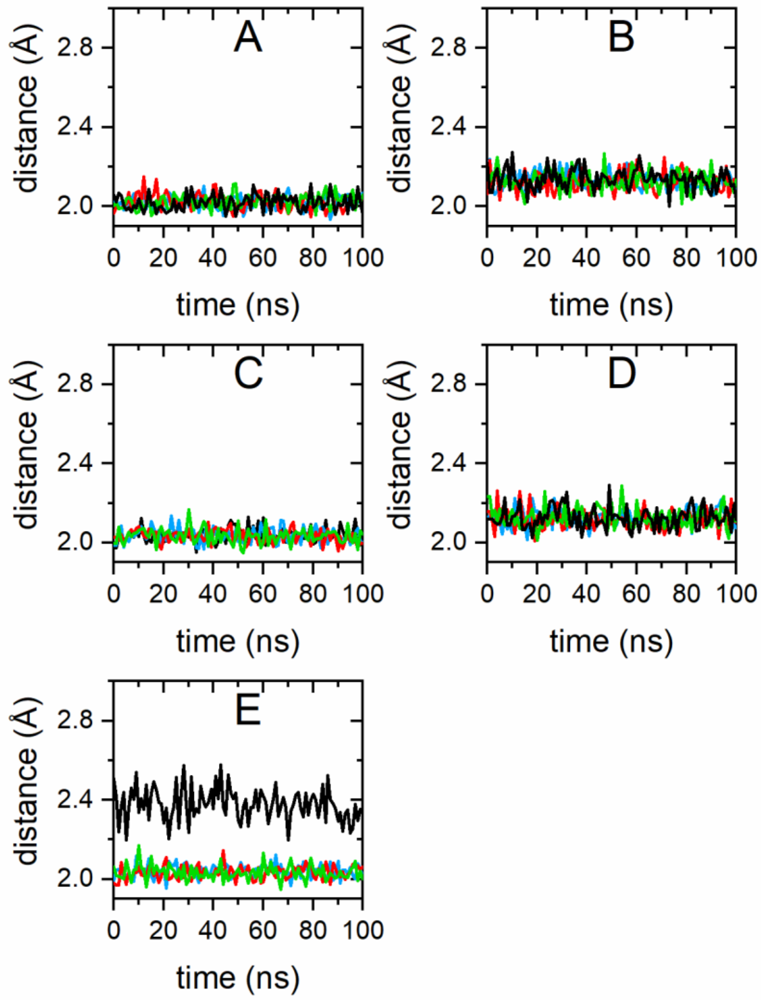

Figure S9. Distances between metal ions and histidine $N^{\varepsilon 2}$ atoms (H61: blue, H69: red, H77: green, H85: black) for simulations of the OR peptide together with: (**a**) a single Cu(II) ion; (**b**) two Cu(II) ions; (**c**) a single Zn(II) ion; (**d**) two Zn(II) ions, and (**e**) two OR peptide molecules with a single Cu(II) ion.
